# Supplementary material for: Conservation aquaculture as a tool for imperiled marine species: Evaluation of opportunities and risks for Olympia oysters, Ostrea lurida
Source: PLoS One. 2021 Jun 21;16(6):e0252810. doi: 10.1371/journal.pone.0252810 (PMC8216563; doi:10.1371/journal.pone.0252810)
Supplement: S1 Table — (PDF) [file pone.0252810.s003.pdf]

S1 Table. Stakeholders who contributed to the development and scoring of the reward vs. risk tables and conservation aquaculture indices.

For each we provide their primary affiliation status, categorized as Grower (G), Manager or Resource Agency representative (M), NGO member (N), Conservation Scientist (S), or Tribal Representative (T). While some fall into multiple categories, we asked each stakeholder to identify the primary role that they assumed to contribute to this project. Stakeholders are arranged in terms of their geographic areas of expertise, approximately from North to South.

Name	Institution	Status
Rhona Govender	Fisheries and Oceans Canada	M
Megan Hintz	Lummi Nation	T
Jamie Donatuto	Swinomish Indian Tribal Community	T
Julie Barber	Swinomish Indian Tribal Community	T
Sarah Grossman	Swinomish Indian Tribal Community	T
Elizabeth Tobin	Jamestown S'Klallam Tribe	T
Jason Haveman	Port Gamble S'Klallam Tribe	T
Tori Cantelow	Point No Point Treaty Council	T
Shannon Miller	Point No Point Treaty Council	T
Tiffany Waters	Global Aquaculture, The Nature Conservancy	N
Betsy Peabody	Puget Sound Restoration Fund	N
Brian Allen	Puget Sound Restoration Fund	N
Jodie Toft	Puget Sound Restoration Fund	N
Brady Blake	Washington Department of Fish and Wildlife	M
Christopher Eardley	Washington Department of Fish and Wildlife	M
Jennifer Ruesink	University of Washington	S
Gifford Pinchot IV	Chelsea Farms Oyster Company	G
John Adams	Sound Fresh Clams and Oysters	G
Dick vander Schaaf	The Nature Conservancy, Oregon	N
Steve Rumrill	Oregon Department of Fish and Wildlife	M
David Couch	City of Arcata, California	M
Gary Fleener	Hog Island Oyster Company	G
Edwin Grosholz	University of California, Davis	S
Chela Zabin	Smithsonian Environmental Research Center	S
April Ridlon	Science for Nature and People Partnership	S
Kerstin Wasson	Elkhorn Slough National Estuarine Research Reserve	M
Danielle Zacherl	California State University, Fullerton	S
Bryce Perog	California State University, Fullerton	S
Jeff Crooks	Tijuana River National Estuarine Research Reserve	M
Julio Lorda	Universidad Autónoma de Baja California	S
