# Supplementary material for: Conservation aquaculture as a tool for imperiled marine species: Evaluation of opportunities and risks for Olympia oysters, Ostrea lurida
Source: PLoS One. 2021 Jun 21;16(6):e0252810. doi: 10.1371/journal.pone.0252810 (PMC8216563; doi:10.1371/journal.pone.0252810)
Supplement: S2 Table — (PDF) [file pone.0252810.s004.pdf]

S2 Table. Scoring guidance for all criteria. Low, Medium and High scores were set individually for each criterion, as described, and assigned scores of 0, 1, and 2 respectively in numeric scoring. If there was no data or high uncertainty, the score was assigned a value of zero in numeric scoring.

Criterion	**Low score (0)**	**Medium Score (1)**	**High score (2)**
1-Recruitment limitation	Recruitment is generally high in this estuary	Some sites or years have recruitment failure	Estuary-wide recruitment failure is common
2-Extinction risk	Adult population stable; likelihood of local extinction very low	Adult population declining but not at immediate risk of local extinction	Adult population declining strongly or entirely absent; at high risk of local extinction from this estuary
3-Mortality	Juveniles and adults have low survival rate (<20% of 10mm+ oysters survive 1 year)	Juveniles and adults have medium survival rates (<50% of oysters survive 1 year)	Juveniles and adults have high survival rates (>50% of oysters survive 1 year)
4-Isolation	Not isolated at all (substantial Olympia oyster populations within 20 km along coastline from this area; OR based on currents or genetics, evidence that this population is quite connected to nearby populations)	Moderately isolated (substantial Olympia oyster population between 20-100 km along coastline from this area; OR based on currents or genetics, evidence that this population has some connectivity to nearby populations	Very isolated (no substantial (>10,000 individuals) Olympia oyster population within 100 km along coastline from this area; OR based on currents or genetics, evidence that this is a largely closed population)
5-Olympia oyster hatchery	Hatchery located >100 km away from estuary	Hatchery located <100 km away, but not on same estuary	Hatchery located on same estuary
6-Safe consumption	Chronic unsafe conditions for shellfish consumption due to high E. coli, pesticides, etc.	Occasional unsafe conditions for shellfish consumption, e.g. due to red tides (Harmful Algal Blooms)	Generally safe conditions for shellfish consumption
7-Olympia oyster harvest	Harvest not allowed	Harvest allowed but certain areas of the estuary where oysters occur are restricted	Harvest allowed anywhere in the estuary where oysters occur
8-Olympia oyster growth	Growth is slower than average for the species	Growth is average	Growth is above average
9-Bivalve farming	No		Yes
10-Olympia oysters in management plan	No		Yes
11-Community engagement - Olympia oysters	No groups currently engaged	1-5 communtiy groups engaged	>5 community groups engaged
12-Community engagement - shellfish	No		Yes
13-Indigenous engagement	No indigenous groups engaged in shellfish restoration or aquaculture	1 or more indigenous groups engaged in shellfish restoration or aquaculture	1 or more indigenous groups engaged in oyster restoration/aquaculture
14-Community shellfish growing	No groups currently engaged	1-2 communtiy groups engaged	> 2 community groups engaged
