# Supplementary material for: Conservation aquaculture as a tool for imperiled marine species: Evaluation of opportunities and risks for Olympia oysters, Ostrea lurida
Source: PLoS One. 2021 Jun 21;16(6):e0252810. doi: 10.1371/journal.pone.0252810 (PMC8216563; doi:10.1371/journal.pone.0252810)
Supplement: Attachment — Submitted filename: PONE-D-20-38508_reviewer_27_12_2020.pdf [file pone.0252810.s007.pdf]

PLOS ONE

Conservation Aquaculture as a Tool for Imperiled Marine Species: Evaluation of Opportunities and Risks for Olympia Oysters

--Manuscript Draft--

**Manuscript Number:**	PONE-D-20-38508
**Article Type:**	Research Article
**Full Title:**	Conservation Aquaculture as a Tool for Imperiled Marine Species: Evaluation of Opportunities and Risks for Olympia Oysters
**Short Title:**	Conservation Aquaculture as a Tool for Olympia Oysters and Other Imperiled Marine Species
**Corresponding Author:**	April D Ridlon, Ph.D.<br>National Center for Ecological Analysis and Synthesis<br>Santa Cruz, CA UNITED STATES
**Keywords:**	conservation aquaculture; oyster, shellfish farming; foundation species; species recovery; species at risk; endangered species; threatened species; decision-support tool; Estuary; restorative aquaculture
**Abstract:**	Conservation aquaculture is becoming an important tool to support recovery of declining coastal species. However, culture of marine organisms for conservation purposes has been carried out infrequently in coastal ecosystems for foundation species, which have significant commercial and cultural value. Olympia oysters (~~<i>~~Ostrea lurida ~~</i>~~) inhabit estuaries in western North America, where they provide key ecosystem functions and services including structured biogenic habitat, trophic support for a broader food web, natural filtration, a commercial fishery and cultural benefits. Olympia oysters have declined precipitously in many estuaries, and aquaculture is one potential tool to support their recovery. However, this tool comes with potential risks as well as rewards which must be assessed to guide aquaculture activities and recovery efforts. Collaboration is essential among user groups with interests ranging from enhancing and restoring existing populations to creating new commercial markets or enhancing culturally important resources. To this end, we characterized the benefits and risks of Olympia oyster aquaculture with a diverse stakeholder team that included restoration practitioners, resource agencies, Native American Tribal representatives, conservation scientists, and shellfish growers. Informed by these trade-offs, we then developed decision-support tools to prioritize among estuaries. Starting with 66 estuaries along the entire range of the Olympia oyster, we used weighted ecological criteria to identify ten priority estuaries where conservation aquaculture is likely to most benefit oyster recovery. We also used other ecological and social criteria to identify which types of aquaculture, including community aquaculture~~/harvest~~ and commercial aquaculture, might be successful to meet the goals of local stakeholders. Our approach, involving a diverse stakeholder team to collaboratively evaluate trade-offs of using aquaculture to support species recovery, and to prioritize conservation aquaculture locations using transparent decision-support tool[illegible] transferable to other systems. This approach can be used elsewhere to seek wi[illegible]n opportunities for expanding aquaculture, including commercial production, at sites where net benefits ~~accrue~~ to both people and the imperiled species.
**Order of Authors:**	April D Ridlon, Ph.D.
	Kerstin Wasson
	Tiffany Waters
	John Adams
	Jamie Donatuto
	Gary Fleener
	Halley Froehlich
	Rhona Govender

Powered by Editorial Manager® and ProduXion Manager® from Aries Systems Corporation

	Aaron Kornbluth
	Julio Lorda
	Betsy Peabody
	Gifford Pinchot
	Steven S Rumrill
	Elizabeth Tobin
	Chela J Zabin
	Danielle Zacherl
	Edwin Grosholz
**Additional Information:**	
**Question**	**Response**
**Financial Disclosure**<br><br>Enter a financial disclosure statement that describes the sources of funding for the work included in this submission. Review the submission guidelines for detailed requirements. View published research articles from *PLOS ONE* for specific examples.<br><br>This statement is required for submission and **will appear in the published article** if the submission is accepted. Please make sure it is accurate.<br><br>**Unfunded studies**<br>Enter: *The author(s) received no specific funding for this work.*<br><br>**Funded studies**<br>Enter a statement with the following details:<br>• Initials of the authors who received each award<br>• Grant numbers awarded to each author<br>• The full name of each funder<br>• URL of each funder website<br>• Did the sponsors or funders play any role in the study design, data collection and analysis, decision to publish, or preparation of the manuscript?<br>• **NO** - Include this sentence at the end of your statement: *The funders had no role in study design, data collection and analysis, decision to publish, or preparation of the manuscript.*<br>• **YES** - Specify the role(s) played.<br><br>* typeset	This work was funded by a David and Lucile Packard Foundation Grant (#2018-68222) to the Science for Nature and People Partnership. Additional support was provided for all co-authors from their respective organizations. The funders had no role in study design, data collection and analysis, decision to publish, or preparation of the manuscript.

Powered by Editorial Manager® and ProduXion Manager® from Aries Systems Corporation

**Competing Interests**<br><br>Use the instructions below to enter a competing interest statement for this submission. On behalf of all authors, disclose any competing interests that could be perceived to bias this work—acknowledging all financial support and any other relevant financial or non-financial competing interests.<br><br>This statement **will appear in the published article** if the submission is accepted. Please make sure it is accurate. View published research articles from *PLOS ONE* for specific examples.<br><br>**NO authors have competing interests**<br><br>Enter: *The authors have declared that no competing interests exist.*<br><br>**Authors with competing interests**<br><br>Enter competing interest details beginning with this statement:<br><br>*I have read the journal's policy and the authors of this manuscript have the following competing interests: [insert competing interests here]*<br><br>* typeset	The authors have declared that no competing interests exist.
**Ethics Statement**<br><br>Enter an ethics statement for this submission. This statement is required if the study involved:<br><br>• Human participants<br>• Human specimens or tissue<br>• Vertebrate animals or cephalopods<br>• Vertebrate embryos or tissues<br>• Field research<br><br>Write "N/A" if the submission does not require an ethics statement.<br><br>General guidance is provided below.	Our work did not involve ‘research on human subjects’; we did not collect information about participating members or study their individual perspectives, and thus did not require Institutional Review Board approval. Instead the input from stakeholders with diverse expertise was synthesized as a collaborative activity with all stakeholders involved in the team effort.

Consult the submission guidelines for detailed instructions. **Make sure that all information entered here is included in the Methods section of the manuscript.**<br><br>**Format for specific study types**<br><br>**Human Subject Research (involving human participants and/or tissue)**<br>• Give the name of the institutional review board or ethics committee that approved the study<br>• Include the approval number and/or a statement indicating approval of this research<br>• Indicate the form of consent obtained (written/oral) or the reason that consent was not obtained (e.g. the data were analyzed anonymously)<br><br>**Animal Research (involving vertebrate animals, embryos or tissues)**<br>• Provide the name of the Institutional Animal Care and Use Committee (IACUC) or other relevant ethics board that reviewed the study protocol, and indicate whether they approved this research or granted a formal waiver of ethical approval<br>• Include an approval number if one was obtained<br>• If the study involved *non-human primates*, add *additional details* about animal welfare and steps taken to ameliorate suffering<br>• If anesthesia, euthanasia, or any kind of animal sacrifice is part of the study, include briefly which substances and/or methods were applied<br><br>**Field Research**<br><br>Include the following details if this study involves the collection of plant, animal, or other materials from a natural setting:<br>• Field permit number<br>• Name of the institution or relevant body that granted permission	
**Data Availability**<br><br>Authors are required to make all data underlying the findings described fully available, without restriction, and from the	Yes - all data are fully available without restriction

Powered by Editorial Manager® and ProduXion Manager® from Aries Systems Corporation

time of publication. PLOS allows rare exceptions to address legal and ethical concerns. See the PLOS Data Policy and FAQ for detailed information.<br><br>A Data Availability Statement describing where the data can be found is required at submission. Your answers to this question constitute the Data Availability Statement and **will be published in the article**, if accepted.<br><br>**Important:** Stating 'data available on request from the author' is not sufficient. If your data are only available upon request, select 'No' for the first question and explain your exceptional situation in the text box.<br><br>Do the authors confirm that all data underlying the findings described in their manuscript are fully available without restriction?	
**Describe where the data may be found in full sentences. If you are copying our sample text, replace any instances of XXX with the appropriate details.**<br><br>• If the data are **held or will be held in a public repository**, include URLs, accession numbers or DOIs. If this information will only be available after acceptance, indicate this by ticking the box below. For example: *All XXX files are available from the XXX database (accession number(s) XXX, XXX.*).<br>• If the data are all contained **within the manuscript and/or Supporting Information files**, enter the following: *All relevant data are within the manuscript and its Supporting Information files.*<br>• If neither of these applies but you are able to provide **details of access elsewhere**, with or without limitations, please do so. For example:<br><br>*Data cannot be shared publicly because of [XXX]. Data are available from the XXX Institutional Data Access / Ethics Committee (contact via XXX) for*	All relevant data are within the manuscript and its Supporting Information files.

researchers who meet the criteria for access to confidential data.<br><br>*The data underlying the results presented in the study are available from (include the name of the third party and contact information or URL).*<br>• This text is appropriate if the data are owned by a third party and authors do not have permission to share the data.<br><br>* typeset	
Additional data availability information:	

Manuscript

Click here to access/download;Manuscript;MS Draft Conservation Aquaculture for Olympia oysters v2.docx

Conservation Aquaculture as a Tool for Imperiled Marine Species: Evaluation of Opportunities and Risks for Olympia Oysters

April D. Ridlon, Science for Nature and People Partnership and National Center for Ecological Analysis and Synthesis, University of California Santa Barbara, 93106 ctenophores@gmail.com, https://orcid.org/0000-0002-4618-9908

Kerstin Wasson, Elkhorn Slough National Estuarine Research Reserve, California, USA 95076 and Ecology and Evolutionary Biology University of California - Santa Cruz, California, USA 95064 kerstin.wasson@gmail.com

Tiffany Waters, Global Aquaculture, The Nature Conservancy, Virginia, USA 22203 tiffany.waters@tnc.org

John Adams, Sound Fresh Clams and Oysters, Washington, USA 98508, johna@skookumpoint.com

Jamie Donatuto, Community Environmental Health Program, Swinomish Indian Tribal Community, Washington, USA 98257, jdonatuto@swinomish.nsn.us

Gary Fleener, Research and Development, Hog Island Oyster Co., California, USA 94940, gary@hogislandoysters.com

Halley Froehlich, Ecology, Evolution & Marine Biology and Environmental Studies, University of California Santa Barbara, California 93106, hefroehlich@ucsb.edu

Rhona Govender, Species at Risk Program, Fisheries and Oceans Canada, British Columbia, Canada V6C3S4, rhona.govender@dfo-mpo.gc.ca

Aaron Kornbluth, Officer, The Pew Charitable Trusts, Washington D.C., USA 20004, akornbluth@pewtrusts.org

Julio Lorda, Facultad de Ciencias, Universidad Autónoma de Baja California, Mexico 22860 and Tijuana River National Estuarine Research Reserve, California, USA 91932, jlorda@uabc.edu.mx

Betsy Peabody, Puget Sound Restoration Fund, Washington, USA 98110, betsy@restorationfund.org

Gifford Pinchot IV, marcopinchot@gmail.com, Chelsea Farms, Washington, USA, 98502

Steven S. Rumrill, Marine Resources Department, Oregon Department of Fish and Wildlife, Oregon, USA 97365, steven.s.rumrill@state.or.us

Elizabeth Tobin, Natural Resources Department, Jamestown S'Klallam Tribe, Washington, USA 98382, etobin@jamestowntribe.org

Chela J. Zabin, Marine Invasions Research, Smithsonian Environmental Research Center, California, USA 94920, zabinc@si.edu

Danielle Zacherl, Department of Biological Science, California State University Fullerton, California, USA 92834, dzacherl@fullerton.edu

Edwin D. Grosholz, Department of Environmental Science and Policy, University of California - Davis, California, USA 95616, tedgrosholz@ucdavis.edu

Abstract

Conservation aquaculture is becoming an important tool to support recovery of declining coastal species. However, culture of marine organisms for conservation purposes has been carried out infrequently in coastal ecosystems for foundation species, which have significant commercial and cultural value. Olympia oysters (*Ostrea lurida*) inhabit estuaries in western North America, where they provide key ecosystem functions and services including structured biogenic habitat, trophic support for a broader food web, natural filtration, a commercial fishery and cultural benefits. Olympia oysters have declined precipitously in many estuaries, and aquaculture is one potential tool to support their recovery. However, this tool comes with potential risks as well as rewards which must be assessed to guide aquaculture activities and recovery efforts. Collaboration is essential among user groups with interests ranging from enhancing and restoring existing populations to creating new commercial markets or enhancing culturally important resources. To this end, we characterized the benefits and risks ~~of~~ Olympia oyster aquaculture with a diverse stakeholder team that included restoration practitioners, resource agencies, Native American Tribal representatives, conservation scientists, and shellfish growers. Informed by these trade-offs, we then developed decision-support tools to prioritize among estuaries. Starting with 66 estuaries along the entire range of the Olympia oyster, we used weighted ecological criteria to identify ten priority estuaries where conservation aquaculture is likely to most benefit oyster recovery. We also used other ecological and social criteria to identify which types of aquaculture, including community aquaculture~~/harvest~~ and commercial aquaculture, might be successful to meet the goals of local stakeholders. Our approach, involving a diverse stakeholder team to collaboratively evaluate trade-offs of using aquaculture to support species recovery, and to prioritize conservation aquaculture locations using transparent decision-

support tools, is transferable to other systems. This approach can be used elsewhere to seek win-win opportunities for expanding aquaculture, including commercial production, at sites where net benefits accrue to both people and the imperiled species.

Introduction

Marine biodiversity and the ecosystem services marine species provide are in decline globally, but it is not too late for these changes to be reversed [1]. Marine foundation species (e.g. kelp, mangroves, corals, oysters) are critical to the structure and resilience of coastal ecosystems, and provide key ecosystem services to human communities around the world [2, 3]. Many marine foundation species have suffered severe population declines due to human activities including overfishing, habitat loss, and climate change (e.g. [4, 5]. Oysters act as foundation species by creating habitat for other estuarine species, and they provide key ecosystem services to human communities around the world [6, 7, 8]. In addition to ameliorating environmental stressors, such as dampening storm surges [9] and improving water quality via increased filtration [10], oysters have provided food for people around the world for millennia, through both harvest of wild populations and aquaculture [11]. However, like many other marine foundation species, oyster populations have declined precipitously: in the United States, there has been an 88% loss in oyster biomass [12] and worldwide, an estimated 85% of oyster reefs have been lost, a figure exceeding the estimated loss of coral reefs [9]. Simultaneously, ever-rising global demand for protein, among other factors, is driving the rapid expansion of shellfish aquaculture [13, 14, 15], including oyster farming, while the restoration of native oyster species and wild oyster beds has become a priority for maintaining the health and ecosystem function of estuaries [16, 17].

The Olympia oyster (*Ostrea lurida*) is native to estuaries from British Columbia, Canada to Baja California, Mexico [18, 19], and a prime example of a foundation species whose populations have drastically declined. Olympia oysters have created habitat for numerous estuarine and coastal species [20, 21], and once supported a vital fishery [22], yet populations are now at 1% of their historic levels and face local extinction in some regions [10]. Thus, while oysters are still present in many estuaries, they no longer form dense, habitat-forming beds in many places. Wild populations of the native oyster are further challenged by the alteration of estuarine habitats, non-native predators, poor water quality, sedimentation, and recruitment [23, 24]. Indeed, a recent synthesis revealed that recruitment limitation is the second biggest challenge to restoration success with Olympia oysters throughout their range, negatively affecting over 70% of all projects (see ArcGIS Story Map summary).

Conservation aquaculture - defined by Froehlich [25] as human cultivation of an aquatic organism for the planned management and protection of a natural resource - is a tool with enormous potential to support both nature as well as people, by providing additional economic and social benefit to communities. While its definition can sometimes include the cross-ecosystem benefits of shifting human food from terrestrial sources towards aquatic, hatchery-raised sources [25], here we focus specifically on its application as a tool to aid the recovery of an imperiled species. Conservation aquaculture emphasizes the importance of ecologically responsible methods to implement, and scientifically rigorous methods to evaluate, the use of aquaculture techniques that purposefully align with conservation goals. Its techniques also specifically seek to minimize the risks sometimes associated with conventional aquaculture. For example, conservation hatchery protocols address risks related to the release of hatchery-reared

organisms, including preserving the genetic diversity of the wild population and minimizing the propagation of invasive species [26, 27]. Conservation aquaculture programs have been successful in supporting the recovery of endangered fish (e.g. white sturgeon: [28]), and invertebrate populations (e.g. white abalone [29]), and aquaculture techniques have successfully supported restoration of declined oyster populations (e.g. eastern oyster *Crassostrea virginica* [30]; European flat oyster *Ostrea edulis* [31]). However, aquaculture has generally been used opportunistically, in sites where existing hatcheries or conservation organizations are located, without range-wide strategic planning to prioritize locations that would benefit imperiled species the most.

Wild Olympia oysters were harvested for millennia by Indigenous people on the west coast of North America [32, 33], but populations declined due to over-harvesting and habitat degradation following European settlement [34, 35]. While cultivation of the Olympia oyster launched a shellfish industry in Washington, efforts at commercial aquaculture of the native oyster remained very limited [22], and today the species is commercially grown only as a specialty product. The Pacific oyster (*Crassostrea gigas)* - a hardy, fast-growing native of east Asia - has instead been the dominant commercially grown oyster on this coast [36], and growers who raise Olympia oysters may be more motivated by an interest in the ecology and heritage of the species than its monetary value. However, there does appear to be an increasing consumer interest and demand for Olympia oysters and the potential for additional growers to create a larger Olympia oyster market with prices equal to or greater than Pacific oysters [37].

The use of aquaculture techniques to support the recovery of Olympia oyster populations began in 2014 in the Puget Sound region, where a range of aquaculture-supported restoration projects are supported by a conservation hatchery partnership between the National Oceanic and Atmospheric Administration (NOAA) and Puget Sound Restoration Fund through the ~~the~~ Kenneth K. Chew Center for Shellfish Research and Restoration. Aquaculture-assisted restoration work has also been employed in central California and Oregon, where estuary-wide recruitment failure is common [23, 38]. Throughout the majority of the Olympia oyster's range, however, very few restoration efforts have utilized aquaculture activities as a component of restoration or enhancement. Until now, there has been no species-level, range-wide approach to strategically planning the expanded use of aquaculture as a tool to support recovery of Olympia oysters.

While conservation aquaculture has potential to support imperiled populations and contribute to re-building stocks, it is likely to be of greatest benefit where reproduction is a key bottleneck to population growth, and where conditions are favorable for survival of juveniles [38]. Aquaculture is only one of many conservation tools, and likely one best used to aid a threatened or endangered species when integrated into a more holistic management strategy (e.g., alongside strict fishing regulations and/or habitat improvement planning~~;~~ [39, 40]. Importantly, conservation aquaculture also comes with potential risks such as the loss of genetic adaptations of the wild population and modification of habitat by the placement of commercial gear or artificial reef structures [41]. These concerns were recently highlighted by efforts to use conservation aquaculture to restore populations of reef building corals [42]. Froehlich [39] emphasized the importance of exploring potential ecological trade-offs associated with

conservation aquaculture and the need to collaboratively work with stakeholders to set shared conservation goals and priorities.

To our knowledge, there has been no thorough evaluation of the rewards versus risks of conservation aquaculture to guide strategic planning for any species on this coast, let alone for Olympia oysters. Before funders, planners, regulators, conservation organizations, or growers move forward with aquaculture initiatives, there is a pressing need to conduct a robust, collaborative evaluation of whether and under what conditions the rewards outweigh the risks. Additionally, detailed spatial analyses are needed to identify the specific locations where conservation aquaculture efforts can be expected to have a high likelihood for success. A transparent and consistent evaluation process is essential to assess sites across the range of the species, so stakeholders can jointly identify those places where aquaculture is most likely to support species recovery and/or enhance local communities. Funders and conservation organizations require assistance to make strategic investments to gain the greatest impact on species recovery, resource management agencies want to know where risks are lowest, and growers want greater confidence in determining how and where their native oyster aquaculture investments can support conservation efforts. Decision-support tools have been developed to inform strategic planning for aquaculture in other regions [43, 44].

Our goal was to conduct strategic planning and develop decision-support tools to identify the use of aquaculture to support recovery of Olympia oysters across the range of the species. We also evaluated human dimensions of aquaculture, including potential for community harvest and commercial production, within the framework of supporting recovery of wild populations. We

designed an inclusive process engaging stakeholders from the beginning, which is much more likely to yield results and reduce conflict [45]. We thus drew from and expanded upon the membership of the Native Olympia Oyster Collaborative (https://olympiaoysternet.ucdavis.edu/), which brings together local stakeholders united by the belief that a coast-wide perspective will lead to better conservation outcomes for the species. Our team of growers, tribal representatives, scientists, and conservation practitioners evaluated the potential of conservation aquaculture for this species, benefitting from both the local expertise represented by participants, and the commitment to coast-wide collaboration. Together, we explored the rewards and risks of aquaculture for this species, identified priority locations for new or expanded investment in this tool, and assessed human dimensions, considering what types of aquaculture projects would be appropriate for those locations. The collaborative approach, with jointly developed criteria applied consistently at a broad geographic scale, can serve as a model for other species where aquaculture may assist in recovery of wild populations.

Methods & Materials

Collaborative Process with Stakeholder Team

We engaged a diverse group of over 30 stakeholders with expertise or interests related to Olympia oyster restoration, conservation, and production of native oysters for local food systems. Members of the stakeholder group were selected from the full geographic range of this species from British Columbia, Canada to Baja California, Mexico, based on their expertise in working with Olympia oysters, with attention to engaging stakeholders with different goals and perspectives. Most were already affiliated with the Native Olympia Oyster Collaborative and were known to our core team as a result of our earlier work establishing this network, though

some were new partners referred to us by members. Our stakeholder group included conservation scientists, restoration practitioners, aquaculture specialists from non-profit organizations and state and federal agencies, marine resource managers, representatives from two Western Washington Treaty Tribes, and commercial oyster growers (S1 Table). To iteratively incorporate expertise with ecological, social, and economic factors, we engaged the stakeholder group in three primary stages: (1) qualitatively identifying and refining the rewards and risks (i.e. trade-offs) for conservation aquaculture of this species, (2) selecting estuaries to evaluate, and (3) creating and scoring the conservation aquaculture indices for each estuary. We communicated through many small virtual meetings and through review of shared on-line documents, and also held discussions with the full group in a two-day virtual workshop. We used collaborative, iterative processes to gather the best available quantitative data, elicit the group's expert opinion, and/or solicit the input of additional local stakeholders where appropriate through focused meetings in particular regions. Our work did not involve 'research on human subjects'; we did not collect information about participating members or study their individual perspectives, and thus did not require Institutional Review Board approval. Instead the input from stakeholders with diverse expertise was synthesized as a collaborative activity with all stakeholders involved in the team effort.

Rewards and Risks of Conservation Aquaculture with Olympia Oysters

Conservation aquaculture provides a suite of rewards and risks that are specific to the species and population under consideration. In March 2020, the stakeholder working group met to explore the wide range of potential rewards and risks associated with conservation aquaculture of

Olympia oysters in western North America. The range of potential rewards and risks was then condensed into broad categories based on the primary beneficiary: (1) Olympia oyster populations, (2) the coastal ecosystem, (3) Tribes / First Nations, and other local community members, (4) conservation practitioners, and (5) commercial growers. Due to the overlap of rewards and risks identified for Tribes / First Nations and local community members, these were later combined into one user group. This list was further condensed to focus on the rewards and risks that were specific to conservation aquaculture, rather than general rewards and risks that could also be applied to oyster restoration.

While the rewards and risks were determined to often have more than one recipient group, one primary recipient group was identified and listed (Tables 2-3). We separately present the rewards and risks relative to our focal beneficiary categories as they can differ according to which entities the project is designed to benefit. For example, if project goals include community engagement, there are different rewards and risks involved than for a project conducted by staff at a conservation organization. Likewise, projects including harvest or commercial production will have different associated rewards and risks than projects focused solely on restoration. Our stakeholders represented and were interested in the full spectrum of these types of project goals, and thus we evaluated the rewards and risks associated with each of them. From the identified rewards and risks, we then formed recommendations for minimizing risks and maximizing rewards.

Estuary Selection

~~Olympia oysters are almost entirely limited to estuaries; there are no large populations along the open coast [18, 22]. Consequently, we~~ focused on evaluating estuaries across the biogeographic range of the species from British Columbia to Baja California (Fig 1). We started with a comprehensive list of US West Coast estuaries [Appendix A in 46]. The stakeholder team then narrowed the list by removing estuaries that are unsuitable for occupation by oysters, such as those known to have extended periods of low salinity or stagnation (i.e. bar-built estuaries, small lagoons that are closed for extensive periods). Team members from British Columbia to Baja California also worked to add appropriate estuaries in those regions to the US list. Two estuaries along the coast ~~are much larger than all others;~~ San Francisco Bay in central California, and the Salish Sea in northern Washington / southern British Columbia~~. For~~ these large estuaries, regional team members delineated sub-basins that could be assessed separately (S1 Fig).

Fig 1. Location of estuaries and their ecological priority index scores. *Names of the numbered estuaries are provided in Fig 2.*

Conservation Aquaculture Indices

We developed 14 criteria to assess the potential value and feasibility of new or enhanced investment in aquaculture to support conservation efforts for the native oyster across its range (Table 1). We focused our assessment on differential benefits, asking where the use of aquaculture may provide a relatively larger conservation benefit or have a greater likelihood of success. This was because it became clear from stakeholder discussions that more information was available on spatial differences in benefits than on risks -- risks such as habitat damage from gear or loss of genetic diversity could occur anywhere, and we lacked clear evidence of such

risks being greater in some places than others. Both ecological and social criteria were included. These criteria were generated and refined from a longer initial list (of about 20 that were initially brainstormed) through an iterative process with the stakeholder team; we dropped those for which there was insufficient information or that upon consideration did not seem critical to the evaluation. The criteria were phrased as questions to clearly articulate what each criterion is addressing. We also jointly crafted a statement of rationale for inclusion of the criteria. Scoring guidelines were developed for each criterion (S2 Table).

Each estuary was assigned a score (2=high, 1=medium, 0=low) for each of the 14 criteria, with higher scores indicating higher priority for aquaculture. We assigned a score of zero to fields with missing data (leading to conservative results - in the future if data are available, these scores may increase). Each estuary was scored first by the expert or experts most familiar with it, using quantitative data (e.g. on recruitment, survival or growth from their own monitoring or publications) where available, their own expertise, and the assistance of additional local experts where appropriate. Next, multiple stakeholders worked in regional teams to review and discuss scores, provide additional relevant information and resources, and reach consensus on scores. Some estuaries are very well-studied with robust data on factors such as recruitment rates or growth rates, while others are poorly characterized. To reflect such differences, each score was annotated with a certainty level: not very certain (*), fairly certain (**), or very certain (***). To document the decision-making process in scoring, the basis for each score was briefly explained, and any relevant citations or internet resources listed; names of stakeholders that conducted the scoring for each estuary were also documented (S3 Table). It should be noted that the evaluation

process described above resulted from a larger collective effort than is reflected in the author list alone (S1 Table).

To identify the places where new or enhanced investment in aquaculture could most benefit Olympia oyster populations, we developed an *ecological priority index* using only the first four ecological criteria. Our team considered the relative importance of each and decided on a weighting scheme that gave recruitment limitation the highest weight, followed by risk of extinction, and finally isolation and post-settlement mortality (Table 1). Index scores were calculated by summing the weighted scores and then dividing by the maximum possible score, so that index scores ranged between 0 (lowest) and 1 (highest). Any estuary with an index of $\geq$0.50 was considered an ecological priority, as this threshold is transparent and indicates that a majority of criteria were met. Estuaries that had no information for the first three criteria (recruitment, extinction risk, mortality) were initially evaluated (S3 Table) but omitted from subsequent analyses because these three criteria are fundamental to any consideration of conservation aquaculture. Our prioritization thus is conservative; in the future if more data are available, more estuaries may emerge as priority sites for conservation aquaculture.

We then developed three additional indices: a *community restoration index*, a *community harvest index*, and a *commercial production index*. At any estuary, a conservation organization or resource management agency could potentially implement small-scale conservation aquaculture if a need to do so is identified, working with a local hatchery to outplant juveniles solely to enhance natural populations. However, we were interested in also addressing the suitability of each estuary for three other types of conservation aquaculture projects: 1) <u>community restoration</u>

(restoration involving hatchery-raised oysters that is primarily driven by local community interests or engagement), 2) community harvest (deployment of hatchery-raised oysters specifically in order to be harvested and consumed by the local community), and 3) commercial production (aquaculture of oysters raised by growers and sold for profit). The indices for all three of these project types included one ecological criterion, post-settlement mortality, because engaging in novel aquaculture endeavors is likely to fail in places with high mortality. Each of the three indices used a different suite of other criteria, weighted differentially (Table 1), as determined by discussion among the stakeholder team. The indices were calculated summing weighted scores and then divided by the maximum possible value (to obtain scores from 0-1) with two exceptions. For community harvest, an estuary automatically received an index score of zero if it had received a zero for safety of shellfish consumption or legality of Olympia oyster harvest, since either of these preclude community harvest. For commercial production, an estuary au[illegible]matically received an index score of zero if it had received a zero for safety of shellfish consumption. Again, estuaries were deemed a high priority for consideration of a particular project type if the index score was ≥0.50.

Table 1. Criteria used in evaluating estuaries for conservation aquaculture. *Each criterion is framed as a question, and the rationale for inclusion is provided. In the final four columns, the weighting of each criterion used to calculate the four indices is shown; if blank, this criterion was not included in the index.*

	Criterion question	Rationale for inclusion in index	Ecological Priority	Community Restoration	Community Harvest	Commercial Production
1	Is **recruitment** limited?	Aquaculture has potential to make biggest positive difference in estuaries with low recruitment.	4			
2	Is the Olympia oyster population at risk of local **extinction?**	Aquaculture has the potential to make the ~~biggist~~ positive difference in estuaries with very low adult populations that are at risk of disappearing.	3			
3	Is post-settlement **mortality** low?	Places with high post-recruitment survival should be prioritized, so return on aquaculture investment is maximimized; avoid sites with high mortality due to high drill predation, freshwater events, etc.	2	2	2	1
4	How **isolated** is this Olympia oyster population?	Estuaries that are geographically and/or genetically isolated and do not have larval exchange from nearby populations are more vulnerable to a potential loss of an entire population, and may benefit more from aquaculture as a tool.	2			
5	Is there a nearby **hatchery** that has produced Olympia oyster spat from local broodstock using conservation protocols?	A hatchery that is in the same estuary will reduce risks associated with shipping spat from greater distances including introducing pathogens, parasites, or non-native foulding species. Conservation protocols are aimed at retaining local genetic adaptations and diversity.		1	1	2
6	Is it **safe to eat** shellfish?	Only areas where water quality allows for safe consumption of can be used for commercial production or community h[illegible]vesting.			3	3
7	Do **regulations allow harvest** of Olympia oysters?	Areas where harvest is allowed should be prioritized for community groups interested in harvest.			2	
8	Is post-settlement **growth** of Olympia oysters high?	High growth rates reduce the length of time between outplant and harvest, relevant to community harvest groups and commercial growers.			1	1
9	Are Olympia oysters or any other species of **bivalves** currently being **farmed** in this estuary?	New or expanded Olympia oyster aquaculture by community groups or commercial growers is facilitated if there is existing infrastructure for farming bivalves, and a track record of safe water quality, harvest, etc.			1	1
10	Is local Olympia oyster restoration/enhancement part of the **management or conservation plan** of any organizations?	Aquaculture investment and permitting is facilitated where native oyster restoration is identified as a priority by multiple organizations.		2		
11	Are there **community** or volunteer groups currently engaged in **Olympia oyster** restoration?	Aquaculture-based restoration is more likely to succeed and positively affect people where there are engaged communities.		2		

12	Are other **community** groups currently engaged in restoration of other species of **bivalve/shellfish**?	Existing groups engaged in restoration with similar species could indicate a capacity for or interest in incorporating Olympia oyster restoration in the future.		1		
13	Are there **Native American Tribes or First Nations** currently engaged in **bivalve/shellfish** restoration or wild harvest?	Indigenous stewardship increases the chances of successful long-term restoration and management of oyster populations, and increases the priority of doing so, to sustain a legacy of cultural practices.		1	1	
14	Are other **community** groups (non-commercial) growing shellfish (oysters or other species) for **harvest?**	Existing groups that grow and harvest shellfish can increase the success of restoration efforts via the stewardship, maintenance and management of oyster beds.			1	1

Results

Rewards and Risks of Conservation Aquaculture with Olympia Oysters

~~Our stakeholder team initially~~ identified 32 different potential rewards and 29 potential risks associated with using conservation aquaculture with Olympia oysters. These were aggregated and refined to 12 rewards and 11 risks that had direct relevance solely to conservation aquaculture projects (e.g. not general rewards that would also be associated with oyster restoration, such as water filtration). Social, cultural, and economic benefits from conservation aquaculture were the most represented ~~positives~~ (67%), compared to the ecological returns (33%). The risks were generally more varied with ecological risks represented more strongly (55%), followed by economic (27%) and then social and cultural (18%). Each benefit or risk was assigned to one of the categories ~~we~~ identified based on the primary beneficiary: Olympia oyster populations, the coastal ecosystem, conservation practitioners, Tribes / First Nations, local community members, and commercial growers.

For Olympia oyster populations, the primary benefit of utilizing conservation aquaculture was the potential to dramatically increase local population numbers fairly rapidly, particularly in recruitment-limited estuaries (Table 2). The primary risks to populations involved genetic concerns associated with using hatchery-reared oysters, including reduced genetic diversity and loss of local adaptations, and the potential for density-dependent emergence and spread of diseases within native oyster populations (Table 3).

While ~~our~~ stakeholder team originally identified many benefits for the coastal ecosystem from Olympia oysters, ~~we did not, identify many~~ that were additive beyond traditional non-aquaculture Olympia oyster restoration. However, two potential rewards of conservation aquaculture to the coastal ecosystem included the reduced introduction of non-native fouling species associated with the culture of native oysters versus introduced oysters, and the benefits of additional (artificial) habitat provided by aquaculture gear, particularly in areas such as unstructured mudflats (Table 2). Potential risks included negative ecological consequences associated with the transfer of oysters from within hatcheries into wild populations and potential negative alterations of the coastal habitat as a result of the use of commercial farming via aquaculture gear (Table 3).

For conservation practitioners, conservation aquaculture may potentially support the management of oyster populations for future climate conditions, since it enables the search for phenotypes that are more resilient to environmental stressors. Conservation practitioners may also benefit from engaging new partners and leveraging the private industry (that are using their own resources for commercial oyster production) for conservation gain (Table 2). Risks included the difficulty in creating conservation-centered best management practices (BMPs) at appropriate

scales for commercial hatcheries, the potential for some in the commercial oyster industry to produce and sell Olympia oysters without adhering to BMPs and protocols that further the goals associated with conservation aquaculture, and a potential for greater competition between industry and conservation groups for funding and/or resources (Table 3).

For Tribes, First Nations, and community groups, a primary benefit of conservation aquaculture projects was the harvest and consumption of a native, locally sourced food in estuaries where wild Olympia oyster populations cannot sustain a harvest. For communities of Indigenous Tribes and First Nations in particular, benefits also included increased access to a traditional food source and the maintenance of social/cultural traditional food practices (Table 2). However, we also identified the potential for lack of early consultation with Tribes and/or First Nations as a primary risk which could lead to the disempowerment of these communities if the management and stewardship priorities for tribally ceded areas are not taken into account prior to the start of a project (Table 3).

Finally, for commercial oyster growers, adding Olympia oysters to complement existing farmed species can provide diversification for the grower’s portfolio, which may be key to continued commercial viability. Additionally, conservation aquaculture of native Olympia oysters can provide benefits related to improving public perceptions of aquaculture and potentially result in increased interest in cultured seafood (Table 2). Risks included Olympia oysters being a generally less profitable and often riskier species to raise due to their slow growth, and the potential of Olympias bio-fouling or settling on other cultivated species (Table 3).

Table 2. Potential rewards of conservation aquaculture of Olympia oysters, organized by primary recipient group. *Only those rewards uniquely associated with conservation aquaculture, not with oyster restoration through any means (e.g. water filtration), are presented.*

Reward to (primary)	*Benefit of Olympia Oyster Conservation Aquaculture*
Olympia Oysters	Scaled or rapidly increased population numbers where there is low recruitment or recruitment failure for populations that are severely declined / at risk of local extinction, and/or isolated from other populations
	Increased genetic diversity in small populations, where genetic bottleneck/allee effects are likely to occur
Coastal Ecosystems	Increased structure and habitat for other fish and marine invertebrates from aquaculture gear
	Reduced introduction of non-native fouling species if fewer non-native oysters are commercially grown, if native oysters become commercially viable
Conservation Practitioners	Ability to manipulate reproduction, test tolerances to environmental conditions (e.g. to search for phenotypes more resilient to climate change effects)
	Leveraging private industry for conservation gain through partnerships with commercial growers
Tribes / First Nations or Local Community Members	Increased community engagement with, knowledge of, and/or stewardship of coastal ecosystem through consumption / harvest of a local food source made possible at a larger scale through aquaculture
	Additional revenue streams (e.g. ancillary downstream businesses) for waterfront and community
	Increased traditional food source benefit
	Maintained social/cultural continuity of traditional food gathering
Commercial Growers	Potential to improve perceptions of aquaculture and/or increase interest in native aquaculture species
	Diversification of, increased income, and increased resilience (climate or other) for grower portfolio

Table 3. Potential risks of conservation aquaculture of Olympia oysters, organized by primary recipient group.

Risk to (primary)	*Risk of Olympia Oyster Conservation Aquaculture*
Olympia Oysters	Reduced genetic diversity of hatchery-raised animals vs. wild population
	Reduced local adaptation and/or plasticity
	Increased disease emergence and/or spread; increase of parasites with increasing population densities
	Increased risk of poaching if hatchery production raises profile of native oysters and increases awareness of wild or restored oyster locations
Coastal Ecosystems	Increased disease or pests spread from Olympia oysters to nearby native species or habitat
	Negative alterations of the natural habitat from aquaculture gear, including increased plastics in the marine environment
Conservation Practitioners	Difficulty in creating BMPs at appropriate scales and/or risk of growers producing or selling Olympia oysters without adhering to BMPs and protocols
	Greater competition between industry and conservation groups for funding and/or resources
Tribes / First Nations or Local Community Members	Disempowerment by not taking into account local community priorities or restricted tribal areas
Commercial Growers	Riskier and less profitable species to raise than non-native species due to slow growth / longer time to harvest
	Increased abundance of Olympia oysters from aquaculture could lead to increased larval production and settlement on other cultivated species, with negative results for both other species and Olympias in being removed from water

Priority Estuaries and Project Types

~~Through our estuary selection process, we~~ identified 66 estuaries as suitable for Olympia oyster populations throughout the species' range along the west coast of North America (with sub-basins of the Salish Sea and San Francisco Bay treated as estuaries). Each of these estuaries was scored for all 14 conservation aquaculture criteria, with scoring rationale and references clearly documented (S3 Table). Of these, 40 estuaries had sufficient data for the first three ecological criteria, which were vital for evaluating the value of conservation aquaculture (Figs. 1-2). The remaining 26 estuaries had insufficient information and were not considered further. Overall, more estuaries at both the northern and southern ends of the distribution were omitted due to limited available data, and the remaining estuaries that were retained in British Columbia and Baja California still had many "unknown" scores for individual criteria. Thus, our results are likely conservative given the data limitations (scores of 0 for missing data will later be increased as data become available).

Of the 40 estuaries with sufficient data, ten estuaries were identified as ecological priorities (Figs. 1-2) for new or increased investment in conservation aquaculture of Olympia oysters. Two of these estuaries (Netarts Bay in Oregon and Elkhorn Slough in California) received the highest possible index score (1.0). Three other estuaries scored quite high (>0.7): Whidbey Basin in Washington and Morro Bay and Carpinteria Marsh in California.

The ten ecological priority estuaries varied in the appropriateness of different project types (Fig 2, S2 Fig). Half of these estuaries ranked highly for community restoration (five estuaries = >.50), and 40% ranked highly for community harvest and commercial production each (four

estuaries = >.50 for each project type). Four of the ecological priority estuaries - all within the central range of the species from San Francisco Bay to Mugu Lagoon - did not rank highly for any of the three project types, and are instead currently best suited for projects led by conservation or resource management organizations.

Fig 2. Conservation aquaculture indices. *Estuaries are arranged from North to South, with province or state abbreviations shown (BCC=British Columbia, Canada; WA= Washington, OR=Oregon, CA=California, USA; BCM= Baja California, Mexico). The names of the ten estuaries that emerged as ecological priorities are shown in bold font; all index scores ≥0.5 are highlighted. The individual scores for each criterion are shown to the right (darker shading represents higher scores; missing data shown in white).*

Discussion

~~Our~~ assessment of conservation aquaculture for Olympia oysters is timely and highlights the need to consider this strategic approach to mitigate the steep declines faced by other marine species globally. We have developed and implemented a transparent and analytical framework (Fig 3) designed to help jumpstart the consideration of conservation aquaculture as a tool to support imperiled species, by linking specific rewards and risks with particular end-user groups. Throughout the planning process, we identified ways in which conservation aquaculture can address both current management challenges (e.g. recruitment failure), and advance forward-thinking strategies for future challenges (e.g. resilience of oysters to the effects of future climate

change scenarios). This type of assessment is needed for other foundation species especially where aquaculture is already being used as a tool to address declines (e.g. reef-building corals).

Fig 3. Conceptual diagram of steps to take in evaluating conservation aquaculture for a new species or region. *The diagram follows a logical chronological flow, but in practice some steps may occur simultaneously or there may be iterative rounds revisiting particular steps. For the first steps, the process should only move forward if the determination of the previous step is affirmative (e.g. only move to step 2 if the species requires restoration, to step 3 if reproduction is deemed limiting, to step 4 if aquaculture is feasible, to step 5 if there is a team, etc.)***.**

Tradeoffs and Risk Mitigation of Conservation Aquaculture

Our collaborative process engaging diverse stakeholders identified the key rewards and risks of conservation aquaculture for Olympia oysters. Below, we discuss these by end user or beneficiary, and make recommendations about minimizing these risks.

Olympia Oyster Populations

Any successful restoration directly benefits the focal species that is being restored. As such, restoration efforts aimed at rebuilding Olympia oyster populations can be beneficial for restoring numbers and distribution towards historical baselines, and in very threatened populations, for preventing local extinction. What makes restoration via conservation aquaculture different from other restoration approaches is the potential to dramatically increase local population numbers fairly rapidly. In recruitment-limited estuaries, this cannot be achieved simply by providing bare settlement substrates, which is the most common restoration approach for Olympia oysters (see

ArcGIS Story Map summary). Supplying thousands of hatchery-raised juveniles can boost recovering populations to a threshold where they can become self-sustaining, as has been accomplished in Fidalgo Bay, Washington [47] and Sequim Bay, Washington (E. Tobin, unpublished data).

The primary risks identified ~~by our stakeholder team~~ involved genetic concerns associated with using hatchery-reared oysters, including reduced genetic diversity and loss of local adaptations [48]. Olympia oysters have locally differentiated population structure [49, 50] and have been shown to exhibit local adaptations for the timing of reproduction [51] and tolerance to salinity [52]. Maintaining such local adaptations when possible is important, so we recommend use of responsible conservation aquaculture protocols such as those developed by Puget Sound Restoration Fund and Washington Department of Fish & Wildlife and used at NOAA's Kenneth K. Chew Center for Shellfish Research and Restoration hatchery, where large numbers of local adults are used as broodstock, and techniques are used to reduce hatchery selection. Considering hatchery conditions is also critical since Olympia oysters have been shown to have strong carryover effects on reproduction relative to environmental conditions [53].

For extremely small populations, especially those facing local extinction, the benefit of increasing the population size may outweigh genetic concerns such as outbreeding depression [41] and introducing genetic diversity into these populations may be beneficial now and in the future as environmental conditions change due to climate change [54]. Our ecological priority index resulted in recommendations for increased use of this tool primarily in estuaries with very

small populations, where potential genetic risks are likely overshadowed by genetic benefits and decreased extinction risk.

The risk of density-dependent emergence and spread of diseases within native oyster populations was also a concern identified by our stakeholder team. Currently, diseases and pathogens do not appear to play a major role in Olympia oyster mortality [55]. However, higher densities in the hatchery or field can increase this risk [56]. Careful monitoring for pathologies, and avoidance of transfers among hatcheries, should be used to manage this risk.

Coastal Ecosystems

Olympia oyster restoration through any means provides a number of benefits to the ecosystem, such as increasing the diversity of benthic-water column coupling and filter feeders [57] and enhancing habitat for other fish and marine invertebrates [21]. While our stakeholder team did not identify many ecosystem-wide benefits exclusive to aquaculture-based restoration, these benefits result from conservation aquaculture projects where it is needed to support populations. One potential conservation aquaculture reward is reduced introduction of non-native fouling species, which include parasites and pathogens associated with culture of non-native species in distant hatcheries, versus native species in local ones. However, this benefit would only accrue if Olympia oysters become commercially viable and production is widespread, resulting in concurrent decreases in aquaculture of non-native species. In addition, the artificial structure provided by aquaculture gear in areas such as unstructured mudflats can provide similar benefits for fish and invertebrates as other ‘artificial reefs’ [8, 58].

However, any aquaculture endeavor involves some level of risk of transferring non-native species, including pathogens and parasites. There are potential negative ecological consequences of conservation aquaculture associated with the transfer of oysters from within hatcheries into wild populations, although disease risks appear modest [55]. In addition, negative alterations of the coastal habitat can occur as a result of the use of commercial farming via aquaculture gear, for example by possibly contributing to microplastics in the estuaries where oysters are grown commercially [59]. Aquaculture gear has the potential to damage particularly sensitive estuarine habitats or create space conflicts with other declining marine foundation species in need of protection, such as seagrasses [5, 60]. We recommend that native oyster culture take place within the existing footprint of commercial operations when possible to minimize negative impacts on natural estuarine habitat that can occur on small spatial scales as the result of shading and increased sedimentation. That stated, there are also likely to be positive effects of aquaculture at larger scales due to increased water column nutrients for seagrasses and structural refugia for fish and invertebrates provided by gear in unstructured mudflat habitats [61, 62, 63]. However, the overall impacts, both positive and negative, and the spatial scales of those impacts, need to be carefully assessed in the deployment of commercial aquaculture gear.

Conservation Practitioners

Conservation aquaculture represents a relatively new and unique tool for practitioners to use for the enhancement or rebuilding of Olympia oyster populations where traditional restoration practices have not been successful or well-funded. As climate change increasingly affects coastal ecosystems, conservation practitioners may also use aquaculture techniques to search for phenotypes more resilient to climate change effects (e.g. those with higher tolerances to changed

or predicted climate-driven environmental conditions), as has been done with other species like corals [64]. Despite its promise, due to the high degree of uncertainty about climate effects on Olympia oysters [65], we recommend further study before we can explicitly support assisted evolution approaches with this species.

Conservation practitioners can also benefit by engaging new partners and leveraging private industry funds and resources from commercial oyster production for conservation purposes. Considerable benefits may be realized despite the limited public funding for conservation in general and for restoration of the Olympia oyster in particular. However, there are risks to conservation practitioners engaging in these new partnerships, including the difficulty in creating conservation-centered best management practices (BMPs) at appropriate scales that can or will be followed in commercial hatcheries, the potential for greater competition between industry and conservation groups for funding and/or resources, and the risk of some in the commercial oyster industry producing and selling Olympia oysters without adhering to BMPs and protocols that further the goals associated with conservation aquaculture.

To address the risks associated with industry partnerships, we recommend that conservation practitioners work with hatcheries that have clear, appropriate conservation practices and protocols in place, and that they work with commercial growers to understand the particular challenges and opportunities faced by hatcheries following these conservation BMPs, to ensure that all parties continue toward shared goals. We also recommend conservation practitioners have transparent conservation-first policies in place regarding their rationales and conditions in which they partner with industries (e.g. [66]).

Tribes, First Nations, and Community Groups

Where populations of oysters cannot sustain a wild harvest, conservation aquaculture projects can provide some specific benefits through the harvest and consumption of Olympia oysters, including connecting people with coastal ecosystems through the enjoyment of a native, locally sourced food. This is evidenced by the recent increase in interest in Olympia oysters in the Pacific Northwest and California as part of the popular “slow food” movement [67]. Rebuilding populations of Olympia oysters can also encourage an increased engagement with, knowledge about, and/or stewardship of coastal ecosystems within communities that may otherwise lack connection with native oysters as a component of West Coast estuaries. For communities in Indigenous Tribes and First Nations in particular, conservation aquaculture projects can increase access to a traditional food source and serve to maintain social/cultural continuity of traditional food practices [68, 69].

However, our working group identified the potential for lack of early consultation with Tribes and/or First Nations as a primary risk which could lead to the disempowerment of these communities if the management and stewardship priorities for tribally ceded areas are not taken into account prior to the start of a project. In addition, further risk ensues when restricted tribal properties are accessed or prioritized for conservation aquaculture by those outside of the community without the appropriate prior consultation and consent from the Tribe or First Nation. We recommend the continuation and expansion of engaging communities in conservation aquaculture projects for restoration and harvest wherever possible. While Indigenous Tribes, First Nations and local communities were combined into one user group in this analysis due to

overlap of rewards and risks identified it should be noted that Indigenous Tribes and First Nations are not stakeholder groups, but sovereign Governments that do not always share the same values, nor are governed by the same regulations, as their non-Indigenous neighboring communities. We recommend that any organization partnering with Indigenous Tribes and First Nations involve those communities directly from the start of the planning of a project and respect tribal access to, applicable legal authorities over, and stewardship of traditional lands. This includes asking permissions to access and conduct projects on land where appropriate, and engaging in a collaborative process to ensure that the community's priorities for resource management, cultural heritage, and stewardship of the land are helping drive the project goals. We also recommend that conservation aquaculture projects that involve harvest include education of the public about not harvesting or disturbing wild Olympia oyster populations that are on restoration sites or in non-harvestable areas.

Commercial Growers

For commercial oyster growers, adding an additional species, such as Olympia oysters, to complement existing farmed species can provide diversification for the grower's portfolio. With the increasing impacts of climate change and an often unpredictable marine environment [70, 71], diversification may be key to continued commercial viability. As with other fisheries, having a more diverse portfolio of oyster species could create additional economic opportunities or buffer against economic loss [72]. For example, a recent outbreak of oyster herpesvirus (OsHV-1) in the *Crassostrea gigas* population in Southern California required the destruction of a season's worth of infected oysters, which could not be sold, representing significant financial losses for growers [73]. Olympia oysters are not as vulnerable to herpes, or many of the other

diseases that plague other commercial species, including *Crassostrea* [55], and show relatively increased resilience to climate effects [65], thus making them ideal to add to the grower's portfolio to buffer against such losses. Also, Olympia oysters provide an entirely different flavor profile than Pacific oysters, and represent a locally-sourced native species, which could provide additional marketing and market differentiation opportunities [37].

Also, engaging in conservation aquaculture of Olympia oysters can provide potential benefits related to public perceptions of aquaculture. While oyster aquaculture differs from other types of fed aquaculture and there is emerging research on the positive ecosystem services that "restorative" commercial shellfish and seaweed aquaculture can provide for water quality, habitat, and climate [8, 61, 74], there remain some negative associations in the US with farmed seafood and marine aquaculture. By focusing on growing a native species and for a stated benefit of increasing native species populations, commercial growers can engage the public under an explicit conservation framework and potentially see increased interest from or improved public perceptions of farmed seafood.

While diversification and perceptions are important, it must be noted that Olympia oysters are generally less profitable, due to their smaller size and longer growing period, and therefore a riskier species for farmers to grow. Also, if Olympia oysters are grown in close proximity to other species, there is the risk that larval overflow could increase "bio-fouling" when Olympia oyster larvae settle on other cultivated species [75], with negative results for both the other cultured species and the Olympias oysters in being removed from the water during harvest.

We recommend engagement of conservation aquaculture by commercial oyster growers in priority areas where: the ecological benefits outweigh the risks; the grower has enough internal financial capacity to support the growing of a species that can take 1-2 years longer to mature and may be initially less profitable than other oyster species; and there is a desire to grow Olympia oysters for both commercial and non-commercial reasons. While the oyster is currently not as profitable as other species, the commercial growers in our expert group that choose to focus on growing and providing Olympia oysters to the public were doing so not only for the market differentiation and potential future profits, but with historical and cultural education benefits, and cultural continuity in mind.

Geographic Prioritization with Conservation Aquaculture Indices

We identified ten estuaries along the entire biogeographic range of the Olympia oyster where new or increased investment in conservation aquaculture is a high priority. Application of the ecological priority index provided a transparent, robust approach to selection of these priority estuaries from ~~the~~ 66 ~~estuaries~~ that were evaluated in a consistent manner. The Native Olympia Oyster Collaborative (https://olympiaoysternet.ucdavis.edu/) was formed to provide such regional perspectives and syntheses, complementing the on-the-ground restoration work which is largely place-based and locally driven, by individual members of the Collaborative. For funders working at a broad spatial scale (e.g. The Nature Conservancy, Pew Charitable Trusts or NOAA), identification of the ten priority estuaries will help direct future grants for conservation aquaculture to the places where the return on investment is highest. For regulatory agencies, a robust process conducted regionally but drawing on local knowledge will help to inform the permitting process for the highest priority estuaries. While our evaluation was conducted across

the full range of the species, the tool we developed can be applied at any scale, for instance for selecting sites within an estuary. We have provided a spreadsheet version (S3 Table) with weighting and formulae so other end users can customize it for their needs. Our tabular approach complements GIS-based decision-support tools developed to prioritize oyster restoration sites [74, 76].

The ecological priority index was designed to prioritize sites where the rewards of aquaculture are likely to greatly outweigh the potential risks. The ten estuaries that emerged as ecological priorities thus represent locations where our stakeholder team can confidently recommend consideration of increased investment in aquaculture as a tool to support native oyster conservation. We weighted recruitment limitation most highly among ecological criteria, since oyster populations where estuary-wide recruitment failure is common (no successful reproduction anywhere in the estuary in many years) stand to benefit the most from enhancement of reproduction through aquaculture. Risk of local extinction due to small or declining population size, and high isolation resulting in lack of larval transport from other populations were also critical determinants of ecological priority. The other key requirement was high post-recruitment survival, since investment in aquaculture is not merited if all outplanted juveniles die.

The two estuaries that scored highest as ecological priorities for conservation aquaculture (Netarts Bay in Oregon and Elkhorn Slough in central California) are places where estuary-wide recruitment failure is common, because current populations are tiny due to past declines, and because larval retention is challenged by low residence time resulting from strong tidal currents

[77]. Both are in areas where genetic analyses suggest isolation is high, with limited connectivity to other populations [50]. Restoration incorporating aquaculture has been attempted in both places [38, 78]. We recommend scaling up these efforts until populations there are self-sustaining, large enough and located in areas of higher residence time so successful recruitment occurs at least in some locations in the estuary in most years.

While we identified ten sites along the coast that represent high priorities for future investment, it is clear that further consideration of the feasibility and potential benefits of conservation aquaculture is required at a local or regional scale before proceeding with new projects. Our decision-support tool used four critical criteria to identify these priority sites, but at a smaller spatial scale, much more information can be used to inform decision-making. Such additional information might lead to local adjustment of prioritization. For instance, Hood Canal in Washington did not emerge as a priority in our index, but conservation aquaculture projects are being considered in the lower portion of the Canal because this location appears to be recruitment limited, other restoration methods may not prove effective in this area, and because there is strong local support for boosting populations. Conversely, Richardson Bay in California did emerge as a priority, but local practitioners have indicated that invasive oyster drills pose a strong threat in many locations, and risks from the predatory drills must be mitigated before proceeding with aquaculture-based restoration at sites with high densities of drills.

The project type indices reveal which approaches are currently possible at each site. At every estuary, a conservation organization or resource management organization could lead a conservation aquaculture project for Olympia oysters. Such projects typically engage community

members as volunteers or in outreach, but are not community-driven -- the design and coordination of the project is led by paid staff from the conservation organization. An example of this is a recent aquaculture-based restoration project at Elkhorn Slough in central California, led by the Elkhorn Slough National Estuarine Research Reserve (Fig 4A).

While the above type of project, led by a conservation organization or resource management agency, is possible anywhere, the ability to implement other types of projects is currently more limited across estuaries. Community restoration depends on leadership by local individuals or groups with strong interests in growing oysters for restoration purposes. For example, in South Puget Sound, tideland owners are passionate about supporting Olympia oysters, and purchase spat to grow juveniles on their tidelands as “shellfish gardens”. In North Puget Sound, the Swinomish Indian Tribal Community is engaged in restoration of oyster habitat as a cultural legacy and to support other species such as salmon (Fig 4B). Restoration led by the community rather than by conservation organizations or resource management agencies appears a viable approach in many places, and currently ranks high as a potential project type at half of the ten priority estuaries (Fig 2).

Community harvest involves local individuals or groups growing oysters specifically for harvest and human consumption. When community harvest occurs in ecological priority areas and contributes to supporting non-harvested components of the population in the estuary through larval spillover, we consider the practice a part of conservation aquaculture. This type of project has promise for Olympia oysters, but is mostly still in the concept stage; we do not know of any examples of successful projects of this type to date. The slower growth rate and lower

availability of hatchery-raised spat makes community harvest projects with the native oyster species more challenging than projects involving the non-native Pacific oyster. Various Tribes in the Puget Sound area and Oregon are interested in restoration of the native oyster for eventual harvest, but since populations are so depleted near Tribal communities, the first step is to rebuild healthy oyster beds, with harvest still an aspirational future goal. Community harvest ranks high as a potential approach to be explored at only four of the ten priority estuaries (Fig 2).

The third conservation aquaculture project type we evaluated is commercial aquaculture. If conducted in ecological priority areas using local broodstock, a nearby hatchery, and conservation protocols, this can contribute to conservation and restoration by generating larvae which support the wild population in the area. Various small-scale growers produce Olympia oysters as a part of their portfolio, and implement appropriate protocols (Fig 4C). Only four of the ten priority estuaries ranked high for implementing this approach (Fig 2). Humboldt Bay and Morro Bay both have commercial aquaculture operations for non-native Pacific oysters; if there were a hatchery nearby producing Olympia oysters from local broodstock, these would also rank as high priorities for Olympia oyster commercial production.

Our analysis revealed strong regional differences in knowledge and capacity for implementing conservation aquaculture projects, which could be decreased through strategic funding and planning efforts. The information available on Olympia Oysters varies substantially across the biogeographic range of Olympia oysters (note varying amounts of missing data in Fig 2). Information needed to assess the benefits or risks of restoration is particularly lacking at both the southern and northern ends of their distribution. In Baja California, additional funding could help

to fill critical data gaps and allow more thorough exploration of restoration needs and potential. In British Columbia, oyster restoration is not a governmental management goal; current management goals are met through implementing the Species at Risk Management Plan for the Olympia Oyster, including surveys to assess shifts in abundance and restrictions on harvest.

Another striking regional pattern is that Indigenous Tribes appear only to be currently engaged in oyster restoration and harvest in Washington and Oregon (dark shading corresponding to a high score for criterion 13 only in top/northern section of Fig 2). Indigenous Tribes and First Nations harvested oysters for millenia along the entire range of the Olympia oyster [32, 33, 79]. Where possible given regional policy and regulations, conservation organizations and resource management agencies could work to engage Indigenous communities that are interested in collaborative efforts to restore native oyster harvests as a traditional food source and cultural practice. However, this could prove challenging where Indigenous people no longer have access to estuaries, or where water quality is so poor as to preclude consumption of shellfish, as is the case in heavily urban and agricultural areas of central and southern California.

A final regional trend relates to distribution of hatcheries, which is also centered in the Pacific Northwest. Indeed, to our knowledge no hatchery has produced Olympia oysters south of Elkhorn Slough in central California -- leaving about 1500 km of the southern range of the species without current hatchery capacity. We recommend that such capacity be advanced in coming years, perhaps by university marine laboratories, particularly somewhere near the three southern California ecological priority sites (Morro Bay, Carpinteria, Mugu Lagoon).

Fig 4. Examples of different approaches to implementing conservation aquaculture with Olympia oysters. *A) Restoration with hatchery-raised juveniles led by a coastal management organization. Staff of the Elkhorn Slough National Estuarine Research Reserve, with community volunteers and partners, assemble stakes with clam shells bearing hatchery-raised juveniles. (Photo: B. Tougher). B) Community restoration. Staff members from the Swinomish Indian Tribal Community work with AmeriCorps volunteers from the Nooksack Salmon Enhancement Association to enhance habitat for Olympia oysters on Swinomish tidelands. (Photo: J. Barber); C) Commercial production and sale of Olympia oysters (Hog Island Oyster Company).*

Conclusions

The new approach and assessment tools developed to evaluate conservation aquaculture for Olympia oysters provide a template that is clearly needed to evaluate the potential use of conservation aquaculture for other species that have undergone declines, and especially in evaluating the tradeoffs between rewards and risks where aquaculture is already being used as a tool to address declines (e.g. reef-building corals). Initiatives are being undertaken globally at many international conservation organizations to engage in conservation aquaculture, and our evaluation can contribute to shaping the use of conservation aquaculture as a tool worldwide. We take an ecological approach that centers on the measurable benefits that conservation aquaculture can uniquely provide to at-risk populations. We incorporate broad stakeholder involvement from the start of the process to develop an explicit risk/reward framework that facilitates decision-making for different goals such as commercial production, community restoration, and community harvest. Our work illustrates how to create and leverage existing partnerships

between agencies, non-profit groups, growers, Indigenous and other communities in order to successfully implement conservation aquaculture. Thus, our Olympia oyster case study provides a model for engaging diverse stakeholders to recommend strategic use of conservation aquaculture where the rewards outweigh risks.

Acknowledgements

We are indebted to our stakeholder team (S1 Table) who contributed to the evaluation of risks and rewards and scoring of estuaries. We thank A. Chang and T. McClintock for specific information associated with estuary selection and the creation of sub-basins within larger complexes. In addition, we thank M. Bitter, M. Camara, J. Moore, D. Hedgecock and B. Valdopalas for help with assessing rewards and risks including population genetics issues and responses to future climate conditions. For initial development of maps and figures, we thank J. Brun, R. Saldivar and M. Klope. This work resulted from the Science for Nature and People Partnership (SNAPP) Conservation Aquaculture Working Group. SNAPP is a partnership of The Nature Conservancy, Wildlife Conservation Society and the National Center for Ecological Analysis and Synthesis at the University of California, Santa Barbara.

References

1. Worm B, Barbier EB, Beaumont N, Duffy JE, Folke C, Halpern BS, Jackson JB, Lotze HK, Micheli F, Palumbi SR, Sala E. Impacts of biodiversity loss on ocean ecosystem services. science. 2006 Nov 3;314(5800):787-90.

2. Angelini C, Altieri AH, Silliman BR, Bertness MD. Interactions among foundation species and their consequences for community organization, biodiversity, and conservation. BioScience. 2011 Oct 1;61(10): 782-789.

3. Mcleod E, Chmura GL, Bouillon S, Salm R, Bjork M, Duarte CM, Lovelock CE, Schlesinger WH, Silliman BR. A blueprint for blue carbon: toward an improved understanding of the role of vegetated coastal habitats in sequestering CO2. Front Ecol Environ. 2011 Dec;9(10): 552-560.

4. Hoegh-Guldberg O, Mumby P, Hooten AJ, Steneck RS, Greenfield P, Gomez E, Harvell CD, Sale PF, Edwards AJ, Caldeira K, Knowlton N, Eakin CM, Iglesias-Prieto R, Muthiga N, Bradbury RH, Dubi A, Hatziolos ME. Coral reefs under rapid climate change and ocean acidification. Science. 2007 Dec 14;318(5857): 1737-1742.

5. Waycott M, Duarte CM, Carruthers TJB, Orth RJ, Dennison WC, Olyarnik S, Calladine A, Fourqurean JW, Heck KL, Hughes AR, Kendrick G, Kenworthy WJ, Short FT, Williams SL. Accelerating loss of seagrasses across the globe threatens coastal ecosystems. Proc Natl Acad Sci. 2009 July 28; 106: 12377-12381.

6. Grabowski JH, Brumbaugh RD, Conrad RF, Keeler AG, Opaluch JJ, Peterson CH, Piehler MF, Powers SP, Smyth AR. Economic valuation of ecosystem services provided by oyster reefs. Bioscience. 2012 Oct 1;62(10): 900-909.

7. Theuerkauf SJ, Morris JA Jr, Waters TJ, Wickliffe LC, Alleway HK, Jones RC. A global spatial analysis reveals where marine aquaculture can benefit nature and people. PLOS ONE. 2019.14(10): e0222282. Available from: https://doi.org/10.1371/journal.pone.0222282

8. Gentry RR, Alleway HK, Bishop MJ, Gilles CL, Waters T, Jones R. Exploring the potential for marine aquaculture to contribute to ecosystem services. Rev Aquac. 2019 Feb 13;12(2): 499-512.

9. Beck MW, Airoldi L, Carranza A, Coen LD, Crawford C, Defeo O, Edgar GJ, Hancock B, Kay MC, Lenihan HS, Luckenbach MW, Toropova CL, Zhang G, Guo X. Oyster reefs at risk and recommendations for conservation, restoration, and management. Bioscience. 2011 Feb;61(2): 107-116.

10. Zu Ermgassen PS, Spalding MD, Grizzle RE, Brumbaugh RD. Quantifying the loss of a marine ecosystem service: filtration by the eastern oyster in US estuaries. Estuaries Coast. 2013 Jan 1;36(1): 36-43.

11. Guo X, Ford SE, Zhang F. Molluscan aquaculture in China. J Shellfish Res. 1999;18(1): 19-31.

12. Zu Ermgassen PS, Spalding MD, Blake B, Coen LD, Dumbauld B, Geiger S, Grabowski JH, Grizzle R, Luckenbach M, McGraw K, Rodney W, Ruesink JL, Powers SP, Brumbaugh R. Historical ecology with real numbers: past and present extent and biomass of an imperilled estuarine habitat. Proc R Soc Lond B Biol Sci. 2012 Sept 7; 279: 3393-3400.

13. Climate change, agriculture and food security. Rome; 2016. Available from: http://www.fao.org/3/a-i6030e.pdf

14. van der Schatte Olivier A, Jones L, Vay LL, Christie M, Wilson J, Malham SK. A global review of the ecosystem services provided by bivalve aquaculture. Rev Aquac. 2020 Feb;12(1) :3-25.

15. Willer DF, Aldridge DC. Sustainable bivalve farming can deliver food security in the tropics. Nat Food. 2020 Jul;1(7): 384-8.

16. Brumbaugh RD, Beck MW, Coen LD, Craig L, Hicks P. A practitioners guide to the design & monitoring of shellfish restoration projects: an ecosystem services approach. Arlington, VA: The Nature Conservancy. 2006. Available from: http://www.oyster-restoration.org/wp-content/uploads/2012/06/tnc_noaa.pdf

17. Fitzsimons, JA, Branigan S, Gillies CL, Brumbaugh RD, Cheng J, DeAngelis BM, Geselbracht L, Hancock B, Jeffs A, McDonald T, McLeod IM, Pogoda B, Theuerkauf SJ, Thomas M, Westby S, zu Ermgassen PSE. Restoring shellfish reefs: Global guidelines for practitioners and scientists. Conserv Sci Pract. 2020 Jun 2;2(6): e18. doi: doi.org/10.1111/csp2.198.

18. Polson MP, Zacherl DC. Geographic distribution and intertidal population status for the Olympia oyster, *Ostrea lurida* Carpenter 1864, from Alaska to Baja. J Shellfish Res. 2009 Mar;28(1): 69-77.

19. Raith M, Zacherl DC, Pilgrim EM, Eernisse DJ. Phylogeny and species diversity of Gulf of California oysters (*Ostreidae*) inferred from mitochondrial DNA. Am Malacol Bull. 2015;33(2): 1-21.

20. Ramsay J. Ecosystem services provided by Olympia oyster (*Ostrea lurida*) habitat and Pacific oyster (*Crassostrea gigas*) habitat; Dungeness crab (*Metacarcinus magister*) production in Willapa Bay, WA [master's thesis]. Oregon State University; 2012. 54 p. Available from: https://ir.library.oregonstate.edu/concern/graduate_projects/fj236741w.

21. Kimbro DL, Grosholz ED. Disturbance influences oyster community richness and evenness, but not diversity. Ecology. 2006;87(9): 2378-2388.

22. Baker P. Review of ecology and fishery of the Olympia oyster, Ostrea lurida with annotated bibliography. J Shellfish Res. 1995;14(2): 501.

23. Pritchard C, Shanks A, Rimler R, Oates M, Rumrill S. The Olympia oyster *Ostrea Lurida*: Recent advances in natural history, ecology, and restoration. J Shellfish Res. 2015;34(2): 259-271.

24. Wasson K, Zabin C, Bible J, Briley S, Ceballos E, Chang A, Cheng B, Deck A, Grosholz T, Helms A, Latta M, Yednock B, Zacherl D, Ferner M. A guide to Olympia oyster restoration and conservation: environmental conditions and sites that support sustainable populations. Elkhorn Slough National Estuarine Research Reserve. 2015 Jun. Available from: www.oysters-and-climate.org

25. Froehlich HE, Gentry RR, Halpern BS. Conservation aquaculture: Shifting the narrative and paradigm of aquaculture's role in resource management. Biol Conserv. 2017 Nov 1;215: 162-168.

26. Flagg TA, Nash CE, Beckman BR, Berejikian BA, Colt J, Dickhoff WW, Fairgrieve WT, Iwamoto RN, Larsen DA, Mahnken CV, Maynard DJ. 1999. A conceptual framework for conservation hatchery strategies for Pacific salmonids. NOAA technical memorandum NMFS-NWFSC ; 38.

27. Crim R, Allen B, Karpoff A, Ryan S, Davis J, Vadopalas B. Olympia Oyster Cultivation For Restoration Part I: Breeding And Hatchery Propagation Techniques. Journal of Shellfish Research. 2015 Aug 1;34(2): 621-621.

28. Schreier AD, Rodzen J, Ireland S, May B. Genetic techniques inform conservation aquaculture of the endangered Kootenai River white sturgeon Acipenser transmontanus. Endangered Species Research. 2012 Jan 18;16(1):65-75.

29. Rogers-Bennett L, Aquilino KM, Catton CA, Kawana SK, Walker BJ, Ashlock LW, Marshman BC, Moore JD, Taniguchi IK, Gilardi KV, Cherr GN. Implementing a restoration program for the endangered white abalone (Haliotis sorenseni) in California. Journal of Shellfish Research. 2016 Oct;35(3):611-8.

30. Brumbaugh RD, Sorabella LA, Johnson C, Goldsborough WJ. Small scale aquaculture as a tool for oyster restoration in Chesapeake Bay. Mar Technol Soc J. 2000 Jan 1;34(1): 79-86.

31. Pogoda B, Brown J, Hancock B, Preston J, Pouvreau S, Kamermans P, Sanderson W, Von Nordheim H. The native oyster restoration alliance (NORA) and the Berlin oyster recommendation: bringing back a key ecosystem engineer by developing and supporting best practice in Europe. Aquat Living Resour. 2019;32(13).

32. Erlandson JM. The role of shellfish in prehistoric economies: a protein perspective. Am Antiq. 1988 Jan 53(1): 102-109.

33. Moss, ML. Shellfish, gender, and status on the Northwest Coast: reconciling archeological, ethnographic, and ethnohistorical records of the Tlingit. Am Anthropol. 1993 Sept 95(3): 631-652.

34. Kirby MX. Fishing down the coast: Historical expansion and collapse of oyster fisheries along continental margins. Proc Natl Acad Sci U S A. 2004 Aug 31; 101(35): 13096-13099.

35. White J, Ruesink JL, Trimble AC. The nearly forgotten oyster: Ostrea lurida Carpenter 1864 (Olympia oyster) history and management in Washington State. Journal of Shellfish Research. 2009 Mar;28(1):43-9.

36. Conte FS, Moore T. Culture of oysters. California's living marine resources: A status report. Sacramento, USA: California Department of Fish and Game. 2001: 500-506.

37. Kaeding L. A resource management analysis of Olympia and Pacific oysters in Puget Sound [master's thesis]. Central Washington University; 2020. 110 p. Available from: https://digitalcommons.cwu.edu/etd/1362.

38. Wasson K., Gossard DJ., Gardner L, Hain, PR, Zabin CJ, Fork S, Ridlon AD, Bible JM, Deck AK, Hughes BB. A scientific framework for conservation aquaculture: a case study of oyster restoration in central California. Biol Conserv. 2020 Oct;250. doi: 10.1016/j.biocon.2020.108745.

39. Froehlich HE, Gentry RR, Rust MB, Grimm D, Halpern BS. Public perceptions of aquaculture: evaluating spatiotemporal patterns of sentiment around the world. PLOS ONE. 2017 Jan 3;12(1): e0169281.

40. Morrison SA. A framework for conservation in a human- dominated world. Conserv. Biol. 2015 Jun;29(3): 960-964.

41. Laikre L, Schwartz MK, Waples RS, Ryman, N. Compromising genetic diversity in the wild: unmonitored large-scale release of plants and animals. Trends Ecol Evol. 2010;25(9): 520-529.

42. van Oppen MJH, Oliver JK, Putnam HM, Gates RD. Building coral reef resilience through assisted evolution. Proc Natl Acad Sci U S A. 2015 Feb;112(8): 2307-2313.

43. Neo ML, Wabnitz CCC, Braley, RD, Heslinga GA, Fauvelot C, Van Wynsberge S, Andrefouet, S, Waters C, Tan ASH, Gomez ED, Costello MJ, Todd PA. Giant clams (Bivalvia:Cardiidae:Tridacninae): a comprehensive update of species and their distribution, current threats and conservation status. Oceanogr Mar Biol. 2017;55: 87-381.

44. Weitzman J. Applying the ecosystem services concept to aquaculture: a review of approaches, definitions, and uses. Ecosyst Serv. 2019 Feb;35: 194-206.

45. Karl HA, Susskind LE, Wallace KH. A dialogue, not a diatribe: effective integration of science and policy through joint fact finding. Environment. 2007 Jan 1;49(1): 20-34.

46. Gleason MG, Newkirk S, Merrifield MS, Howard J, Cox R, Webb M, Koepcke J, Stranko B, Taylor B, Beck MW, Fuller R, Dye P, Vander Schaaf D, Carter J. A conservation assessment of West Coast (USA) estuaries. Arlington, VA: The Nature Conservancy. 2011. 65 p.

47. Dinnel, P. Restoration of the Olympia Oyster, Ostrea lurida, in Fidalgo Bay and Cypress Island. Year Sixteen Report. Skagit County Marine Resources Committee. 2018 Dec. Available from: http://www.skagitmrc.org/media/25803/Final%20Native%20Oyster%20Report%20%202018.pdf

48. Camara MD, Vadopalas B. Genetic aspects of restoring Olympia oysters and other native bivalves: balancing the need for action, good intentions, and the risks of making things worse. J Shellfish Res. 2009;28(1): 121-145.

49. Stick DA. Identification of optimal broodstock for Pacific Northwest oysters [dissertation]. Corvallis, OR: Oregon State University; 2011. Available from: https://ir.library.oregonstate.edu/concern/graduate_thesis_or_dissertations/g732dc75z

50. Silliman KE, Bowyer TK, Roberts SB. Consistent differences in fitness traits across multiple generations of Olympia oysters. Sci Rep. 2018:8(1): 1-8.

51. Barber JS, Dexter JE, Grossman SK, Greiner CM, Mcardle JT. Low temperature brooding of Olympia oysters (Ostrea lurida) in northern Puget Sound. J Shellfish Res. 2016 Aug;35(2): 351-357.

52. Bible JM, Sanford E. Local adaptation in an estuarine foundation species: implications for restoration. Biol Conserv. 2016 Jan 1;193: 95-102.

53. Spencer LH, Venkataraman YR, Crim R, Ryan S, Horwith MJ, Roberts SB. Carryover effects of temperature and pCO 2 across multiple Olympia oyster populations. Ecol Appl. 2020 Apr;30(3): e02060.

54. Sgrò CM, Lowe AJ, Hoffmann AA. Building evolutionary resilience for conserving biodiversity under climate change.Evol Appl. 2011 Mar;4(2): 326-37.

55. Moore JD, Juhasz CI, Robbins TT. A histopathology survey of California oysters. California Fish and Game Report. 2011 Mar 1;97: 68-83.

56. Fisheries & Oceans Canada. Management plan for the Olympia oyster (*Ostrea conchaphila*) in Canada. Species at risk act management plan series. Ottawa: Fisheries & Oceans Canada. 2009: vi-31.

57. Ruesink JL, Feist BE, Harvey CJ, Hong JS, Trimble AC, Wisehart LM. Changes in productivity associated with four introduced species: ecosystem transformation of a 'pristine' estuary. Mar Ecol Prog Ser. 2006;311: 203-215.

58. Costa-Pierce BA, Bridger, CJ. The role of marine aquaculture facilities as habitats and ecosystems. In: Stickney RR, McVey JP, editors. Responsible marine aquaculture. New York: CABI Publishing; 2002. pp. 105-144.

59. Kazmurik TN, Kzmiruk VD, Bendell LI. Abundance and distribution of microplastics within surface sediments of a key shellfish growing region of Canada. PLOS ONE. 2018;13(5): e0196005.

60. Dumbauld BR, Ruesink JL, Rumrill SS. The ecological role of bivalve shellfish aquaculture in the estuarine environment: a review with application to oyster and clam culture in west coast (USA) estuaries. Aquaculture. 2009;290: 196-223.

61.Alleway HK, Gillies CL, Bishop MJ, Gentry RR, Theuerkauf SJ, Jones R. The ecosystem services of marine aquaculture: valuing benefits to people and nature. Bioscience. 2019;69(1): 59-68.

62. Tallis HM, Ruesink JL, Dumbauld B, Hacker S, Wisehart LM. Oysters and aquaculture practices affect eelgrass density and productivity in a Pacific Northwest Estuary. J Shellfish Res. 2009; 28(2): 251-161.

63. Ferriss BE, Conway-Cranoss LL, Sanderson BL, Hoberecht L. Bivalve aquaculture and eelgrass: a global meta-analysis. Aquaculture. 2019;498: 254-262.

64. Morikawa MK, Palumbi, SR. Using naturally occurring climate resilient corals to construct bleaching-resistant nurseries. Proc Natl Acad Sci U S A. 2019 May 19;116(21): 10586-10591.

65. Hollarsmith JA, Sadowski JS, Picard MM, Cheng B, Farlin J, Russell A, and Grosholz, ED. Effects of seasonal upwelling and runoff on water chemistry and growth and survival of native and commercial oysters. Limnol Oceanogr. 2020 Feb;65(2): 224-235.

66. The Nature Conservancy (TNC). Working with companies: principles of corporate engagement [internet]. 2020. Available from: https://www.nature.org/en-us/about-us/who-we-are/how-we-work/working-with-companies/corporate-principles/.

67. Slow food manifesto: international movement for the defense of and the right to pleasure [internet]. 1989. Available from: https://slowfood.com/filemanager/Convivium%20Leader%20Area/Manifesto_ENG.pdf

68. Augustine S, Dearden P. Changing paradigms in marine and coastal conservation: a case study of clam gardens in the Southern Gulf Islands, Canada. Can Geogr. 2014 Mar 7;58(3): 305-314.

69. Stephenson J, Berkes F, Turner NJ & Dick J, Biocultural conservation of marine ecosystems: Examples from New Zealand and Canada. Indian J Tradit Know. 2014,13(2): 257.

70. Meyer GR, Lowe GJ, Kim E, Abbott CL, Johnson SC, Gilmore SR. Health status of Olympia oysters (Ostrea lurida) in British Columbia, Canada. J Shellfish Res. 2010;29(1): 181-185.

71. Waldbusser GG, Gray, MW, Hales B, Langdon CJ, Haley BA, Gimenez I, Smith SR, Brunner EL, Hutchinson G. Slow shell building, a possible trait for resistance to the effects of acute ocean acidification. Limnol Oceanogr.. 2016;61(6): 1969-1983.

72. Cross SF, Flaherty M, Byrne A. Diversification of aquaculture in North America. In: Harvey B, Soto D, Carolsfeld J, Beveridge, M, Bartley DM, editors. Planning for aquaculture diversification: the importance of climate change and other drivers. FAO. 2017: 93-110.

73. Friedman CS, Reece KS, Wippel BJ, Agnew MV, Dégremont L, Dhar AK, Kirkland P, MacIntyre A, Morga B, Robison C, Burge CA. Unraveling concordant and varying responses of oyster species to Ostreid Herpesvirus 1 variants. Science of The Total Environment. 2020 Oct 15;739:139752.

74. Theuerkauf SJ, Eggleston DB, Puckett BJ. Integrating ecosystem services considerations within a GIS-based habitat suitability index for oyster restoration. PLOS ONE. 2019;14(1): e0210936. Available from: https://doi.org/10.1371/journal.pone.0210936

75. Townsend CH. Report of observations respecting the oyster resources and oyster fishery of the Pacific coast of the United States. US Commission of Fish and Fisheries, Report of the Commissioner for 1889-1891. 1893; 343-372.

76. Puckett, BJ, Theuerkauf SJ, Eggleston DB, Guarjardo R, Hardy C, Gao J, Luettich RA. Integrating larval dispersal, permitting, and logistical factors within a validated habitat suitability index for oyster restoration. Front Mar Sci. 2018;5(76): 1-14.

77. Wasson K, Hughes BB, Berriman JS, Chang AL, Deck AK, Dinnel PA, Endris C, Espinoza M, Dudas S, Ferner MC, Grosholz ED. Coast- wide recruitment dynamics of Olympia oysters reveal limited synchrony and multiple predictors of failure. Ecology. 2016 Dec;97(12): 3503-16.

78. Archer PE. Re- establishment of the native oyster, *Ostrea conchaphila*, in Netarts Bay, Oregon, USA [master's thesis]. Oregon State University; 2008. 64 p. Available from: https://ir.library.oregonstate.edu/concern/graduate_thesis_or_dissertations/zs25xd822

79. Laylander D, Iversen D. SDI-4553, Major shellfish genera and prehistoric change on the San Diego county coast. Pacific Coast Archaeological Society Quarterly. 2008;39(4): 39-48.

Supporting Information

S1 Table. Stakeholders who contributed to the development and scoring of the reward vs. risk tables and conservation aquaculture indices.

S2 Table. Scoring guidance for all criteria.

S3 Table. Detailed version of conservation aquaculture scores and indices in Excel, including scores and rationale for them for all 66 estuaries, and including formulae for calculating indices, so that users can add new sites, new data, or change weightings using this template.

S1 Fig Boundaries among sub-basins of the two largest estuaries on this coast. Numbers are the same as used in Figs. 1-2 in the main paper; additional estuaries that were initially considered but were excluded from the index calculations due to insufficient data are labeled with letters.

S2 Fig. Map of project type indices at the ten sites scoring highest on the ecological priority index. Bar heights correspond to site specific values in Fig 2 in main paper.

Fig 1

Click here to access/download;Figure;Fig1.tif

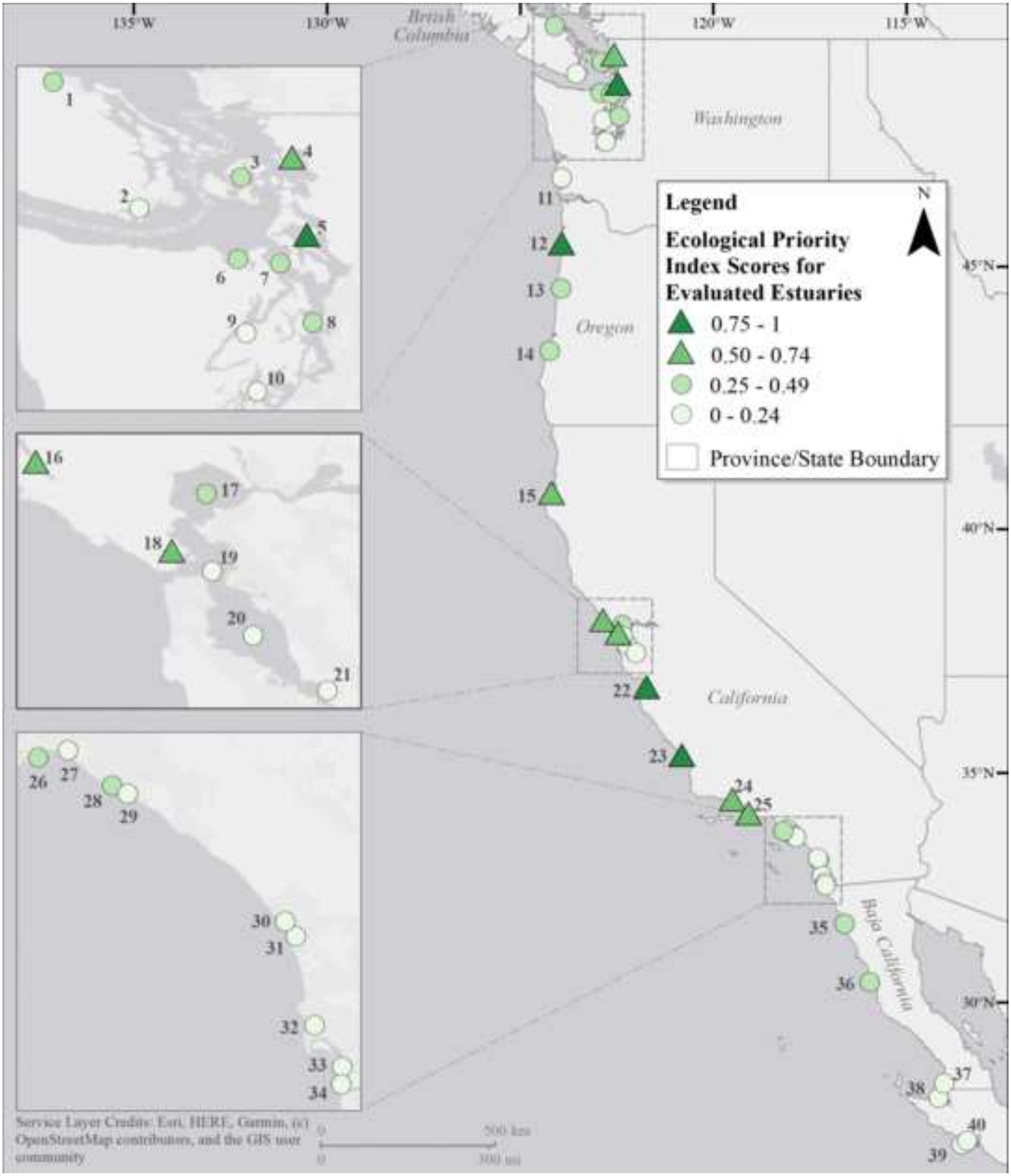

Fig 2

Click here to access/download;Figure;Fig2.tif

Province/State	Map number	ESTUARY	ECOLOGICAL PRIORITY	Community Restoration	Community Harvest	Commercial Production
BCC	1	North Juan de Fuca Basin	0.09	0.28	0.00	0.00
	2	West Strait of Georgia	0.45	0.06	0.00	0.28
WA	3	**Northern Puget Sound**	0.64	0.89	0.75	0.78
	4	**Whidbey Basin**	0.77	0.67	0.00	0.83
	5	Discovery, Sequim Bays	0.27	0.78	0.83	0.94
	6	Admiralty Inlet	0.36	0.56	0.71	0.78
	7	San Juan Islands	0.36	0.44	0.71	0.78
	8	Central Puget Sound	0.27	0.89	0.79	0.89
	9	Hood Canal	0.00	0.56	0.71	0.83
	10	South Puget Sound	0.09	0.67	0.79	0.89
	11	Willapa Bay	0.09	0.22	0.63	0.56
OR	12	**Netarts Bay**	1.00	0.67	0.67	0.78
	13	Yaquina Bay	0.36	0.44	0.50	0.44
	14	Coos Bay	0.36	0.39	0.46	0.44
CA	15	**Humboldt Bay**	0.50	0.00	0.50	0.44
	16	**Tomales Bay**	0.50	0.56	0.75	0.83
	17	SF North/San Pablo Bay	0.27	0.56	0.00	0.00
	18	SF Central Bay North	0.09	0.56	0.00	0.00
	19	SF Central Bay South	0.18	0.44	0.00	0.00
	20	SF South Bay	0.18	0.44	0.00	0.00
	21	**SF Richardson Bay**	0.59	0.44	0.00	0.00
	22	**Elkhorn Slough**	1.00	0.67	0.00	0.00
	23	**Morro Bay**	0.82	0.00	0.42	0.44
	24	**Carpinteria Marsh**	0.73	0.00	0.00	0.17
	25	**Mugu Lagoon**	0.55	0.22	0.00	0.00
	26	Long Beach Harbor	0.27	0.00	0.00	0.00
	27	Alamitos Bay	0.09	0.22	0.00	0.00
	28	Santa Ana River	0.27	0.33	0.21	0.17
	29	Newport Bay	0.09	0.44	0.00	0.00
	30	Agua Hedionda Creek	0.00	0.00	0.00	0.00
	31	Batiquitos Lagoon	0.00	0.00	0.00	0.00
	32	Mission Bay	0.00	0.00	0.00	0.00
	33	San Diego Bay	0.09	0.33	0.00	0.00
	34	Tijuana Estuary	0.18	0.00	0.00	0.00
BCM	35	Estero de Punta Banda	0.32	0.00	0.50	0.44
	36	Bahia San Quintin	0.32	0.00	0.50	0.44
	37	Laguna Manuela	0.23	0.00	0.50	0.44
	38	Ojo de Liebre	0.23	0.00	0.50	0.44
	39	La Bocana	0.23	0.00	0.42	0.33
	40	El Coyote	0.23	0.00	0.50	0.44

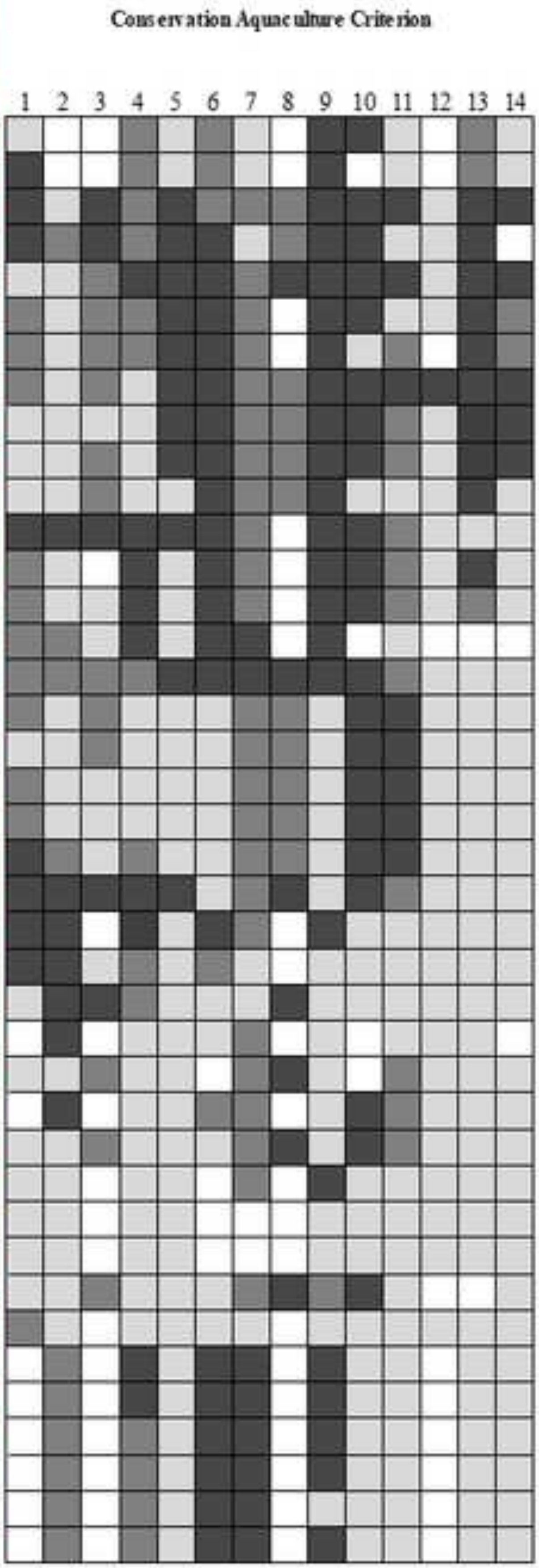

Fig 3

Click here to access/download;Figure;Fig3.tif

EXPLORE NEEDS AND FEASIBILITY

1) Determine whether species requires restoration	Have populations declined? Are numbers lower than historically?
2) Assess whether reproduction is limiting population growth	Is recruitment a main factor in low population growth at least in some places?
3) Evaluate whether aquaculture is feasible	Has this or related species been raised in hatchery? Could it be?
4) Assemble experts and stakeholders with which to complete all subsequent steps	Aquaculture experts, conservation scientists, communities benefiting from services

IDENTIFY AND WEIGH TRADE-OFFS

5) Brainstorm with stakeholders and experts on potential risks and rewards	Draw on personal experience, literature with similar species, etc.
6) Prioritize most important trade-offs	Group and evaluate them
7) Decide whether rewards outweigh risks for this species	(At least in some places)

PRIORITIZE LOCATIONS AND PROJECT TYPES

8) Identify possible locations for projects	Where it has declined, or where conditions are suitable for future
9) Develop criteria for evaluating net benefit to the species of aquaculture in each location	Incorporate risks and rewards identified above
10) Develop criteria for assessing suitability of different project types	Production by conservation organization vs. commercial growers, etc.
11) Design indices that combine information from multiple criteria	Summary calculation may weight some criteria more than others
12) Score all locations for the criteria and indices	Priority locations and suitable project types emerge

Fig 4 Click here to access/download;Figure;Fig4.tif

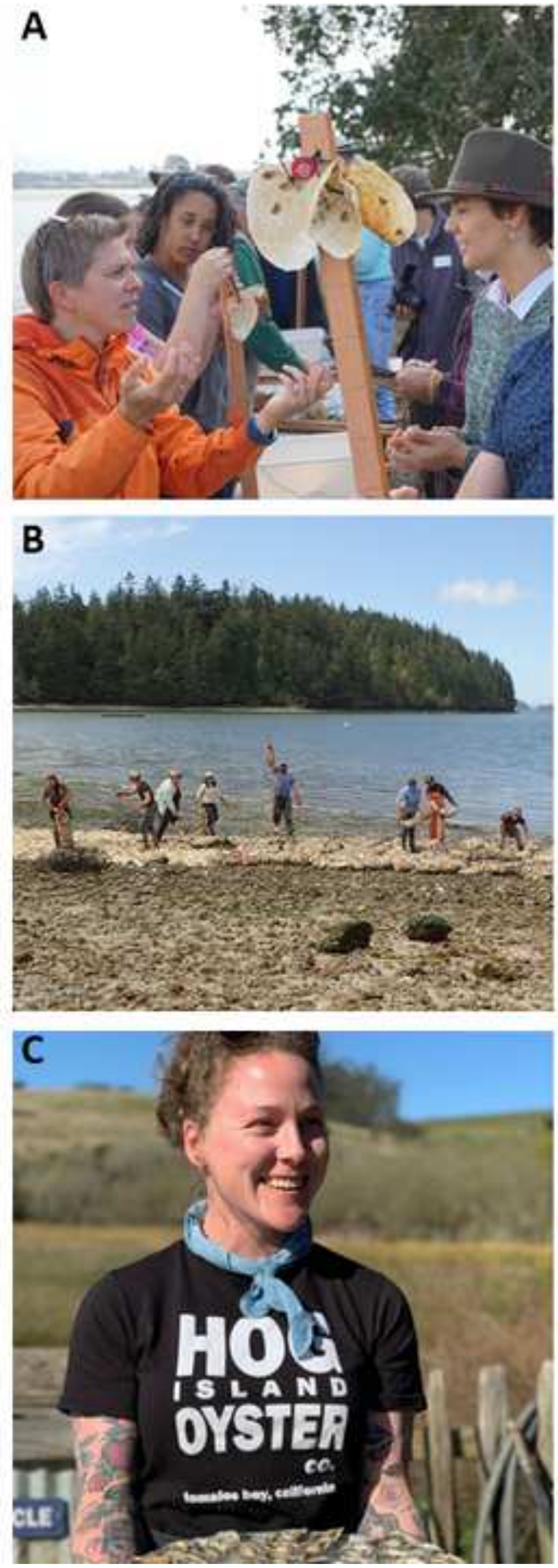

S1 Fig

Click here to access/download
Supporting Information
S1_Fig.tif

S2 Fig

Click here to access/download
Supporting Information
S2_Fig.tif

S1 Table

Click here to access/download
Supporting Information
S1_Table.pdf

S2 Table

Click here to access/download
Supporting Information
S2_Table.pdf

S3 Table

Click here to access/download
Supporting Information
S3_Table.xlsx
