# Supplementary material for: Conservation aquaculture as a tool for imperiled marine species: Evaluation of opportunities and risks for Olympia oysters, Ostrea lurida
Source: PLoS One. 2021 Jun 21;16(6):e0252810. doi: 10.1371/journal.pone.0252810 (PMC8216563; doi:10.1371/journal.pone.0252810)
Supplement: Attachment — Submitted filename: comments to Authors.docx [file pone.0252810.s008.docx]

**GENERAL COMMENTS**

The manuscripts presents findings from a study that aimed at assessing potential risks and rewards associated with *a conservation aquaculture as an important tool* to support recovery of declining species particularly Olympia oysters (*Ostrea lurida*). The study also came up with key recommendations for establishing conservation aquaculture. Generally the manuscript is fairly well written. However, there are few areas that needs specific attention before is recommended for publication in the journal as follows:

1) Authors are advised to strongly minimize personalization of the work such as we, our study etc! Although it gives a good story but this is more of impressing than communicating. In science we write to communicate facts.
2) Authors are trying to put too many or multiple things within a single sentence which is in most cases may confuse readers. “A sentence should express a single thought or proposition”. Authors should avoid long and rambling sentences.
3) It is advisable to have section where key recommendations from this study can be found.

**TITLE**

It is okay. However, inclusion of a scientific name of Olympia oyster at this stage could add a lot of value.

**ABSTRACT**

It is okay. Authors may consider inclusion of a sentence that provides some principal findings of this study.

**INTRODUCTION**

**Lines 39 to 42**: Start with giving worldwide figure and then those from United States of America. Also, provide the estimated figure for loss of coral reefs.

**MATERIALS AND METHODS**

1) **Lines I61 to 164**: In the **S1 Table**, to my opinion, multiple categories could have their own specific category just like the way it is for say those who fall specifically to Grower (G), Manager or Resource Agency representative (M), NGO member (N), Conservation Scientist (S), or Tribal Representative (T). Those who fall under multiple categories should be treated separately and their opinion provided. This information should also be indicated somewhere in lines 161 to 164.
2) **Line 193:** Please refine the sentence to make it clearer.
3) **Line 194** (for Tables 2 & 3) compared with line 224 (Table 1). Tables and figures should be cited serially by starting with 1 (Start with Table 1 or Figure 1). Thus the arrangement could be Table 2 changed to Table 1; Table 3 changed to Table 1 and finally Table 1 changed to Table 3 and cited accordingly for consistency purposes.
4) **Line 202 and part of line 203**: This is information plus the references can be moved to somewhere in the introductory part. In this section just tell the reader the area focused by the study.
5) **Lines 215 to 216**: Can Authors show the criteria used to delineate the sub-basins?
6) **Lines 334 to 338** (many others): It is fortunate that authors are good in English language. Otherwise, most of the sentences are very long! Imagine a sentence of 63 words!! Not recommended for scientific writings!

**RESULTS**

Please see some track changes in this section in the main manuscript.

**DISCUSSION**

1) **Line 436**: Unpublished data cannot be accessible by readers. As such it is not recommended in scientific publications.
2) **Lines 443 to 447**: Very long sentence. Readers may not enjoy reading long sentences! Again a reference to those protocols or even a website link where those institutions can found could be very useful to readers.
3) **Line 460 and several other places**: The use of phrases such as “…..our stakeholders” is monotonous in this manuscript. Authors can frame this work in such a way that such phrases only remain in the Materials and Methods section. Then, make sure that whatever is presented in the results and discussed in the discussion section reflects the findings from stakeholder’s views. I believe this I believe this is doable.
4) **Lines 510 to 516:** How can 77 words be in a single sentence!! Readers cannot follow! There is a time where readers may lose the flow contents!
5) **Lines 557 to 559**: Something is missing in this sentence. Please make it clear.

**CONCLUSION**

The conclusion is somewhat okay. An opening sentence summarising the overall objectives of the study followed by key take home messages would add a lot of value. Authors should strictly provide the main conclusion as a result of carrying this study. Please try to reduce the “hows” here i.e the way the study was undertaken.
